# Detecting *In-Situ* oligomerization of engineered STIM1 proteins by diffraction-limited optical imaging

**DOI:** 10.1371/journal.pone.0213655

**Published:** 2019-03-25

**Authors:** Prasanna Srinivasan

**Affiliations:** La Jolla Institute of Allergy and Immunology, La Jolla, California, United States of America; University of Debrecen, HUNGARY

## Abstract

Several signaling proteins require self-association of individual monomer units to be activated for triggering downstream signaling cascades in cells. Methods that allow visualizing their underlying molecular mechanisms will immensely benefit cell biology. Using enhanced Green Fluorescent Protein (eGFP) complementation, here I present a functional imaging approach for visualizing the protein-protein interaction in cells. Activation mechanism of an ER (endoplasmic reticulum) resident Ca^2+^ sensor, STIM1 (Stromal Interaction Molecule 1) that regulates store-operated Ca^2+^ entry in cells is considered as a model system. Co-expression of engineered full-length human STIM1 (ehSTIM1) with N-terminal complementary split eGFP pairs in mammalian cells fluoresces to form ‘puncta’ upon a drop in ER lumen Ca^2+^ concentration. Quantization of discrete fluorescent intensities of ehSTIM1 molecules at a diffraction-limited resolution revealed a diverse set of intensity levels not exceeding six-fold. Detailed screening of the ehSTIM1 molecular entities characterized by one to six fluorescent emitters across various in-plane sections shows a greater probability of occurrence for entities with six emitters in the vicinity of the plasma membrane (PM) than at the interior sections. However, the number density of entities with six emitters was lesser than that of others localized close to the PM. This finding led to hypothesize that activated ehSTIM1 dimers perhaps oligomerize in bundles ranging from 1–6 with an increased propensity for the occurrence of hexamers of ehSTIM1 dimer units close to PM even when its partner protein, ORAI1 (PM resident Ca^2+^ channel) is not sufficiently over-expressed in cells. The experimental data presented here provide direct evidence for luminal domain association of ehSTIM1 monomer units to trigger activation and allow enumerating various oligomers of ehSTIM1 in cells.

## Introduction

**Fig 1:**
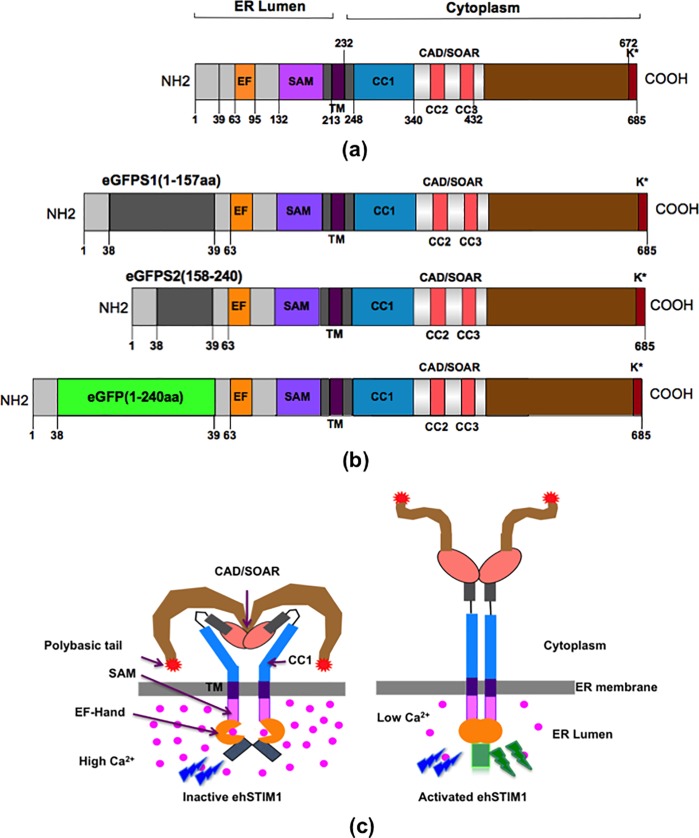
Schematic of hSTIM1 structure and its activation mechanism. (a) Key amino acid positions and functional domains of hSTIM1 protein. The N-terminal luminal domain has a Ca^2+^ binding EF hand and a sterile alpha motif (SAM). The C-terminal cytoplasmic fragment has two coiled-coils (CC1 and CC2) with high predictive probability and a putative coiled-coil (CC3). The CRAC activation domain (CAD) or STIM1-ORI1 activating region (SOAR) and the polybasic (K*) tail are critical for the gating Ca^2+^ channel, ORAI1 (b) Three engineered hSTIM1 constructs namely, eGFPS1-hSTIM1, eGFPS2- hSTIM1 and eGFP-hSTIM1 were used for functional imaging experiments. eGFPS1 and eGFPS2 represents 1-157aa and 158-240aa of an eGFP molecule respectively. eGFP-hSTIM1 is a positive control for the complementation assay. (c) Schematic of the self-assembly of split eGFP pairs by luminal domain association of eGFPS1-hSTIM1 and eGFPS2-hSTIM1 after Ca^2+^ drop inside ER lumen. In the resting inactive state, the luminal domains are positioned far apart preventing self-assembly of split eGFP pairs and hence cannot fluoresce upon excitation by blue light. A drop in the ER Ca^2+^ concentration causes the luminal domains of engineered eGFPS1-hSTIM1 and eGFPS2-hSTIM1 to approach thereby self-assembling the complimentary eGFP split pairs. Matured and functional eGFP formed after self-assembly fluoresces upon excitation by blue light. Activated ehSTIM1 releases the CAD/SOAR domain to extend for gating ORAI1.

Specific interaction between STIM1 and Calcium Release-Activated Calcium (CRAC) channel, ORAI1 invokes calcium signaling in cells by store-operated calcium entry (SOCE). STIM1-ORAI1 coupling machinery regulates Ca^2+^ influx in cells, which is critical for controlling several short and long-term functions [1,2]. STIM1 is a single pass ER membrane resident protein with a Ca^2+^ sensor inside the ER lumen and an extendable actuator in the cytoplasm. In the inactive state, the actuator domains fold back to form a compact structure. During SOCE, the luminal domains of STIM1 sense Ca^2+^ drop inside the ER lumen and transmit the information to the cytosolic actuator domains. The cytosolic fragment then takes an elongated conformation eventually leading to oligomerization and translocation to ER-PM junctions for gating ORAI1 to elicit Ca^2+^ influx. Fig 1A shows a schematic representation of amino acid positions and various domains of human STIM1 (herein referred to as ‘hSTIM1’). Structural details about intramolecular interactions [3], functional mutants [4], models for transmembrane helical packing [4,5], structure of EF-SAM in Ca^2+^ bound state [6], the plausible mechanism of Ca^2+^ sensing by EF-SAM [7] and the molecular structure of CAD/SOAR [8] domain have almost fully unravelled the conformational dynamics of STIM1 from the inactive to active state.

In spite of this wealth of structural information, direct in-situ experimental evidence to confirm the association of luminal domains of hSTIM1 are fragmentary. Earlier works [5, 9] confirmed the approaching of STIM1 luminal domains under a low Ca^2+^ condition invitro. However, in-situ cell-biology evidence [10–12] based on FRET (Förster Resonance Energy Transfer) assays were used to characterize the association of STIM1 luminal domains. While these results provide an indication for the approaching of STIM1 luminal domains in cells, it is sufficiently not persuasive to confirm the EF-hand mediated hSTIM1 signaling in cells. This is mainly attributed to the fact that FRET occurs over a range of a Förster distance (<10) nm. Secondly, it is impossible to get a distance measurement from the apparent FRET efficiency reported in these works without extensive calibration and even if one is obtained it can greatly underestimate the actual distance [13] between luminal domains. The apparent FRET efficiency reported can therefore only serve as a qualitative metric to indicate the approaching of luminal domains if the expression levels of the proteins are nominal. Furthermore, it is not possible to enumerate the oligomeric species of activated STIM1 in-situ in such a set-up. Hence, experiments concerning luminal domain association of STIM1 proteins and in-situ characterization of STIM1 oligomers have not been clarified unambiguously yet.

To overcome these limitations of the in-situ FRET assay, this study presents an alternative functional imaging approach using eGFP complementation. Using eGFP (enhanced GFP) complementation method discussed elsewhere [14], here I present direct evidence for luminal domain association of an engineered hSTIM1 (ehSTIM1) upon depletion of ER Ca^2+^ stores. Self-association of the luminal domains of ehSTIM1 monomer units causes activation leading to oligomerization and translocation to ER-PM junctions as reported previously [11]. This serves as the first step for information transduction by ehSTIM1 across the ER membrane to its cytosolic domains. Furthermore, in vitro [9] and in-situ [10–12] experiments already confirmed that activated STIM1 proteins oligomerize which are subsequently translocated to ER-PM junctions [15]. However, enumeration of different oligomeric species and their spatial distribution in cells is not known yet which can bring new insights into the STIM1 signaling. Here, I report activated ehSTIM1 dimers translocated to ER-PM junction can exist in several oligomeric states ranging from 1–6 dimers and these are characterized by intensity quantization at diffraction-limited optical resolution.

## Results and discussion

Complementation of split GFP reporters (and its variants) was already used to visualize protein-protein interaction in various eukaryotic systems including mammalian cells. Association of several split GFP pairs was known to fluoresce [16–19] but their fluorescence dictated by the self-assembly and maturation of split pairs apparently vary with the split architecture [20]. This technique involves expressing complementary eGFP domains fused to the interacting proteins, which fluoresce when the interacting partners are stably bound. Full-length hSTIM1 molecules when carefully engineered using complementary eGFP reporters (ehSTIM1) fluoresce upon self-association of luminal domains driven by low Ca^2+^ condition inside ER lumen. This simple approach will confirm that luminal domains interact together if ehSTIM1 has to be activated. Secondly, fluorescent intensity provides a direct readout to enumerate ehSTIM1 oligomerization states after a drop in ER Ca^2+^ concentration.

The interaction between coiled-coil domains can bring complementary eGFP split pairs (1-157aa and 158-240aa) together for self-assembly and maturation. This was already demonstrated in vitro and in-situ using anti-parallel leucine zippers [21]. Recently, Cysteine cross-linking experiments [5] confirmed that juxtaposition of the cytosolic coiled-coil domain 1 (CC1) of hSTIM1 coordinates with the approaching of transmembrane domains in as prepared ER membranes under a low Ca^2+^ condition. Hence engineered hSTIM1 molecules with complementary split eGFP pairs fused to the N-terminal luminal domain are likely to self-assemble when ER lumen Ca^2+^ concentration is reduced. Weak interaction between the binding partners [22] is known to influence favorably the self-assembly of split eGFP pairs and its maturation. Given the weak interaction between the luminal domains of ehSTIM1, activated conformation might be frozen irreversibly due to self-assembly and maturation of eGFP split pairs after the depletion of ER Ca^2+^ stores.

Fig 1B shows schematics of the different engineered hSTIM1 constructs used for molecular imaging experiments (See S1 Fig and S1 Table for eGFP-hSTIM1 genetic and amino acid sequences). A fully functional eGFP-hSTIM1 was used as a positive control for the imaging experiments. Engineered hSTIM1 constructs with N-terminal eGFP split pair–eGFPS1-hSTIM1 (1-157aa of eGFP) and eGFPS2-hSTIM1 (158-240aa of eGFP)) were cloned by traditional molecular biology techniques using eGFP-hSTIM1 as the starting material (See Materials and Methods). Fig 1C illustrates the molecular mechanism of eGFP complementation when the luminal domains of eGFPS1-hSTIM1 and the eGFPS2-hSTIM1 are brought together during activation. When Ca^2+^ is bound to the EF-SAM domains of complementary hSTIM1 molecules, their luminal domains were positioned far apart that no fluorescent emission occurs when excited by blue light. However, when the Ca^2+^ concentration inside the ER lumen is reduced, the luminal domains of eGFPS1-hSTIM1 and eGFPS2-hSTIM1 interact forming a fully functional eGFP molecule that fluoresces upon excitation by blue light.

**Fig 2:**
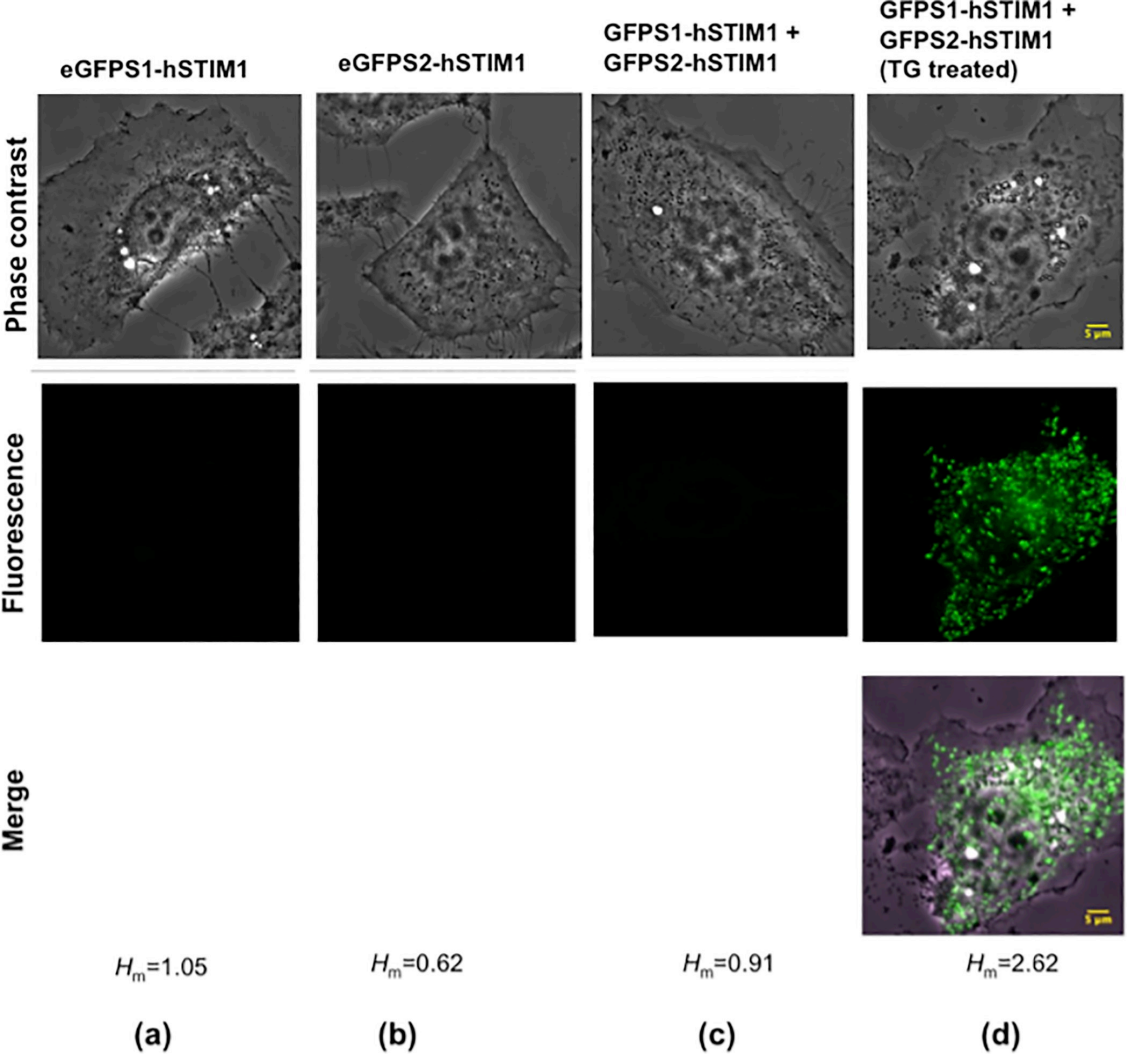
Luminal domain of engineered STIM1 self assemble upon reducing ER calcium concentration. Phase contrast and projected laser confocal micrographs of HeLa cells expressing different engineered hSTIM1 constructs. HeLa cells expressing (a) eGFPS1-hSTIM1, (b) eGFPS2-hSTIM1, and (c) co-expressing eGFP-S1-hSTIM1 and eGFP-S2-hSTIM1. (d) Depletion of Ca2+ from the ER stores of a cell co-expressing ehSTIM1 molecules after addition of Thapsigargin (1*μ*M final concentration). Intramolecular dimerization of luminal domains of engineered hSTIM1 molecules creates a functional eGFP domain that fluoresces when excited by blue light. Increase in the average Shannon Entropy, *H*_m_ correlates positively with the increase in the fluorescence, which is a proxy of ehSTIM1 dimerization / Oligomerization. The scale bar shown is 5*μ*m.

Live single HeLa cell expressing various engineered hSTIM1 constructs was imaged at a diffraction-limited resolution using a laser confocal microscopy (See Materials and Methods). Fig 2A–2D show phase-contrast and fluorescent (projected z-steps with ~300nm step size) confocal micrographs of HeLa cells expressing eGFPS1-hSTIM1, eGFPS2-hSTIM1 and eGFPS12-hSTIM1 (referred henceforth as ‘ehSTIM1’) under untreated and thapsigargin (TG) treated conditions. TG is a Sarco Endoplasmic Reticulum Ca^2+^ ATPase (SERCA) pump inhibitor that decreases the efficiency of Ca^2+^ pumping into the ER lumen. This, in turn, reduces the Ca^2+^ concentration inside the ER lumen. For each construct, a phase contrast, eGFP fluorescence, and their superimposition were shown. It is visibly evident from the micrographs that both eGFPS1-hSTIM1 and eGFPS2-hSTIM1 constructs do not fluoresce on their own (or may be weakly fluorescent insensitive to the detector) when overexpressed separately. This result agrees with an earlier finding which, reported that the chosen complementary eGFP split pairs did not fluoresce on their own even at concentrations >100*μ*m [21]. When the complementary constructs were co-expressed, the baseline fluorescent signal due to luminal domain interaction if any is negligibly small that is not visibly apparent (Fig 2C). However, when the Ca^2+^ level drops inside the ER lumen after TG treatment, the cells fluoresce with an average fluorescent intensity ten-fold greater than that of the untreated condition. This result confirms that Ca^2+^ drop inside the ER lumen directly results in the interaction between ehSTIM1 luminal domains (See S2 Fig for the representative images obtained from another experiment). This interaction brings the complementary split eGFP pairs possibly at a farthest approaching distance ≤ 4nm (width of eGFP beta barrel reported previously [23]), otherwise, a fluorescent eGFP domain cannot be formed. Secondly, activated ehSTIM1 form puncta at ER-PM junction (Fig 2D) as reported by several earlier studies. As a positive control, TG treatment of cells expressing eGFP-hSTIM1 also forms the characteristic fluorescent puncta (See S3 Fig). An earlier study reported that STIM1 puncta colocalize with that of ORAI1 when ORAI1 is sufficiently co-expressed [24]. In contrast, the data presented here confirm that activated ehSTIM1 form puncta even if ORAI1 is maintained at the native expression level. It is highly unlikely that endogenous ORAI1 colocalize at all the sites of the activated ehSTIM1 population given the orders of difference in the respective protein expressions.

Confirming the self-association of a large molecule like hSTIM1 using complementary eGFP split pair is indeed a novel chemical biology method because this approach so far has been applied to study the interaction of small biomolecules. Several lines of evidence were presented to confirm the interaction of ehSTIM1 luminal domains by correlating fluorescent intensities and Shannon information entropy, *H* values. Increase in *H* signifies the diversity in the population of allowable pixel intensities because Ca^2+^ bound ehSTIM1 proteins do not fluoresce efficiently (See Materials and Methods and S4 Fig for the image histogram). Methods for computing *H* using fluorescent intensities were already reported in several works including my recent one [25]. S5A Fig shows the average eGFP fluorescent intensities for different engineered constructs and treatment conditions. As observed before, the highest value of the average intensity corresponds to TG treated cells co-expressing ehSTIM1 and this is ten-fold greater than that obtained for any other conditions. S5B Fig shows that the effective number of gray levels encoding the mean Shannon information, 2^Hm^~6 increases three-fold more than the baseline value (~2^1^ = 2) as the average fluorescence increase by ten-fold after TG treatment. A greater number of ehSTIM1 dimerize/oligomerize neither at the footprint nor at the top surface of the cell but somewhere intermediate between these extremes (See S5C Fig). Such a large difference in fluorescent signal positively correlating with *H*_m_ suggests that ehSTIM1 tends to become more ordered as fluorescent puncta. This orderliness cannot be attained by chance because ehSTIM1 activation has a less likelihood of occurrence on its own without a significant drop in Ca^2+^ concentration inside the ER lumen. This is clearly evident from the negligible background fluorescence observed under untreated condition. Registering eGFP fluorescence and comparing the diffraction-limited fluorescent intensities at several locations locally will allow unraveling the distribution of different ehSTIM1 oligomers spatially.

**Fig 3:**
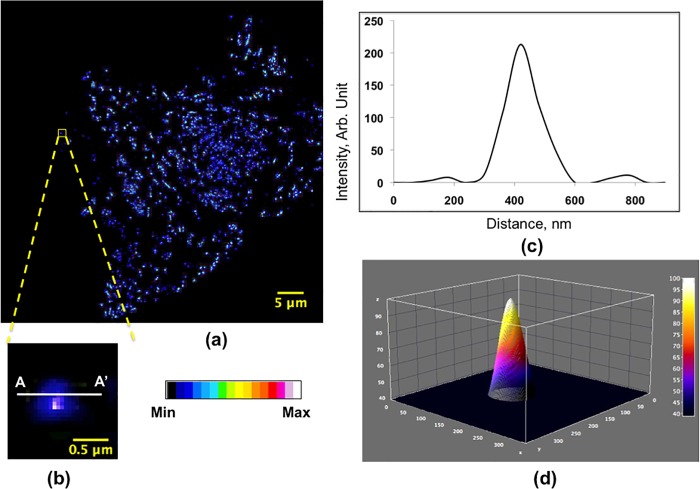
ehSTIM1 puncta formed by depletion of ER calcium at a diffraction-limited resolution. (a) Heterogeneity (blue to white) in the localization of various ehSTIM1 oligomeric species forming puncta at a diffraction-limited resolution. (b) A certain number of emitters localized as a diffraction-limited fluorescent spot in isolation. The asymmetric blue contour surrounding the central ‘hotspot’ can be attributed to the orientation of the dipole moment of fluorophore within the focal plane; (c) Representative intensity trace of the diffraction-limited fluorescent spot across the in-plane cross-section AA’. The intensity trace shows the first two optical modes of the fluorescent spot. (d) Representative 3D PSF of the isolated fluorescent spot. The ‘hotspot’ at the peak confirms the localization of emitters at the centre of the three- dimensional PSF.

Perhaps the most striking result is the observation of ehSTIM1 puncta at a diffraction-limited resolution because this allows enumerating oligomers of an ehSTIM1 population at ER-PM junctions. Fig 3A shows a population of ehSTIM1 molecules spatially localized at varied densities close to the PM of a representative cell (footprint) shown previously (Fig 2D). Heterogeneity in the intensity distribution signifies diversity in the number of ehSTIM1 molecules localized in the vicinity of the PM. Evaluation of the intensity profiles of several individual clusters revealed a few isolated diffraction-limited fluorescent spots as in Fig 3B (See Materials and Methods and S6 Fig). Similar spots exist in the other z-steps as well, predominantly at the extremities (See S7 Fig). The intensity distribution shown in Fig 3B has an asymmetric pattern around the central hotspot. Theoretical models [26] attribute this pattern to translation of angular dipole moment from the mean focal distance during z-traverse although the diffusion of the molecule is negligible while imaging these spots (See Materials and Methods).

It is well known that the ultimate resolution of an optical microscope can be modeled theoretically by Ernst Abbe criterion [27] which is slightly improved in a confocal set up due to narrowing of the point spread function (PSF) to the size of the pinhole (See Materials and Methods). Fig 3C and 3D show the 2D and 3D intensity profiles of the diffraction-limited fluorescent spot represented by Fig 3B across the cross-section AA’. The 2D lateral intensity trace clearly reveals the first two optical modes [28] of the PSF. Two characteristic measurements can be directly obtained from the PSF that allows quantifying the apparent number of emitters and their lateral separations when present in population; the peak intensity and the lateral full-width half maximum (*FWHM*_L_). Full-width half maximum (FWHM) of a PSF is a suitable metric to quantify the spatial precision of single molecule localization accurately [29]. The experimentally evaluated *FWHM*_L_ (~140 nm) obtained from Fig 3C agrees excellently with the theoretically calculated *FWHM*_L_ ~ 139.7 nm [30] (See Materials and Methods). Irrespective of the number of colocalized emitters, the PSF would look similar with peak intensity scaling proportionately with the number of emitters when isolated. Hence, it is impossible to determine the number of emitters using a single peak PSF for an isolated spot. However, if individually digitized PSFs of emitters are laterally spaced apart at least by a distance of *FWHM*_L_, it is possible to estimate the number of emitters spaced laterally by applying intensity quantization rule. This is true only when the separation in axial localization exceeds the *FWHM*_A_ ~386 nm. Hence the clusters are chosen so that their fluorescent signals are not influenced by the signals of the localized neighboring clusters if any from adjacent focal planes for the chosen z-step of ~ 300 nm.

**Fig 4:**
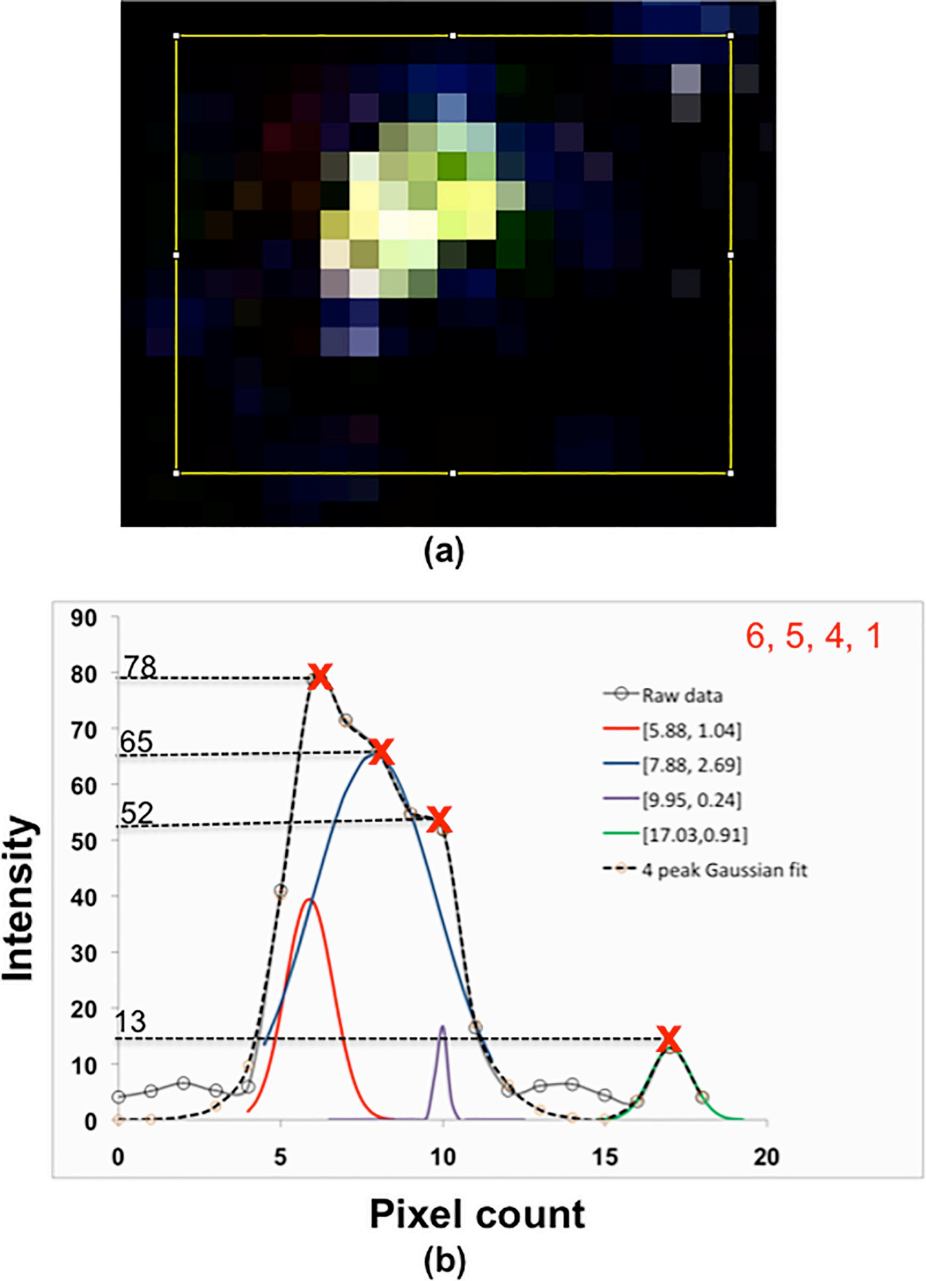
Representative average intensity traces of spatially localised emitters from frame 7. (a) Illuminated pixels representing fluorescent emitters localized at the focal plane inside a 19x15 pixel window. (b) Average intensity trace along horizontal direction spanning 19 pixels. The discretely quantized peak intensities at 13, 52, 65 and 78 arb units should correspond to ehSTIM1 entities comprising 1, 3, 5 and 6 emitters. These emitters are either one of the pseudo representation of colocalized ehSTIM1 oligomers or a true single oligomeric species (See Fig 5). The spatial location of a different number of emitters and the precision of localization was obtained by fitting a four peak Gaussian curve. The mean and standard deviation of each peak for different number of emitters was represented as [*μ*,σ].

**Fig 5:**
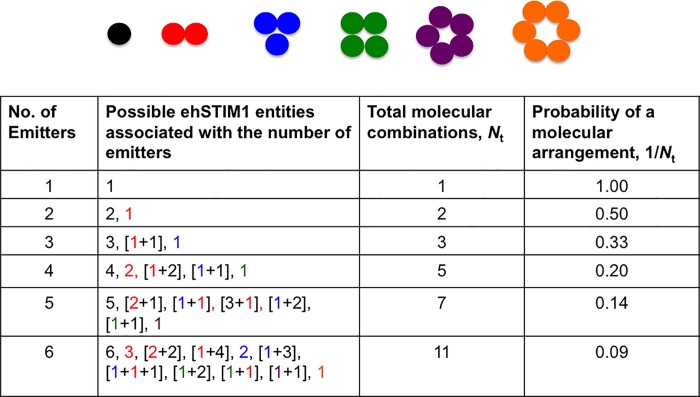
Pseudo and true representations of ehSTIM1 molecular entities for different number of emitters. Various possible pseudo representations are due to colocalization of multiple oligomeric species within 61 nm in different combinations. Both pseudo and true representations are color coded in accordance to the respective colors of various oligomers shown pictorially. Each circle represents a single emitter i.e. one ehSTIM1 dimer unit.

In principle, the intensity of diffraction-limited spots linearly increases with the number of emitters [31] and this relation can be used to quantify the number of ehSTIM1 dimer units in oligomers spatially. This technique was already used to enumerate E-Cadherin oligomers on cell-surface [32]. Applying quantization of the peak intensities of PSFs separated laterally at least by *FWHM*_L_ as a criterion, multiple oligomers of ehSTIM1 dimers localized spatially can be characterized in a chosen cluster. Fig 4A shows illuminated pixels of ehSTIM1 molecules within a 19 × 15 pixels window (< 2 *μ*m size) in frame 7. The plot shown in Fig 4B is the corresponding average intensity trace of the pixels along the horizontal direction. The peaks of the intensity trace are approximately located at 78, 65, 52 and 13 arbitrary units. From these values, it is evident that a highest common factor (13 arb. units) perhaps should correspond to a single emitter (i.e. an ehSTIM1 dimer). Assigning any greater number of emitters for ~13 arb. units are inadmissible given the maximum background intensity of ~ 6.5 arb. units. Using this calibration, the peaks of the intensity trace should, therefore, correspond to 6, 5, 4 and 1 emitters respectively. Since the size of each pixel is ~ 61 nm (an order greater than the size of a single STIM1 dimer unit [3]), it is impossible to quantify the molecular arrangement of ehSTIM1 based on the number of emitters except for the case of a single emitter. For instance, a molecular entity with six emitters can either be attributed to a pseudo representation caused by co-localization of ehSTIM1 oligomers ranging 1–5 dimer units in ten different combinations or to a true representation of a single hexamer (See Fig 5). Similar combinations exist for other entities with the different number of emitters as well. The local peak intensities separated by a distance of > 180nm exceeds *FWHM*_L_ of the PSF of a diffraction-limited spot (~140 nm). Curve fitting of the raw intensity trace using four Gaussian functions (See Materials and Methods) enabled to predict the spatial locations for different number of emitters, i.e. mean and standard deviation of the four peaks ([μ,σ] = [5.88,1.04], [7.88, 2.69], [9.95, 0.24], [17.03, 0.91]). The predicted Gaussian profile for 5 emitters has *FWHM*_L_ > 140nm and these 5 ehSTIM1 dimers are unlikely to exist as a single oligomer of five dimer units. Perhaps it is a pseudo representation caused by co-localization of multiple oligomers within 140 nm in one of the seven different ways. In contrast, the predicted Gaussian profiles for 6, 4 and 1 have *FWHM*_L_ varying between ~ 140–180 nm and there is a finite probability (as shown in Fig 5) that these quantized intensity levels could be a true representation with their respective number of ehSTIM1 dimer units. Using this screening method, emitters localized laterally at different z depths were identified and ranked. Representative PSFs depicting localization of the different number of emitters resolved spatially by *FWHM*_L_ at different z-depths reveal discernable and identifiable oligomeric species in a majority of the clusters (See frames 5, 10 and 11 in S8A and S8B Figs and frame 7 in Fig 4A and 4B). A few intensity traces with *FWHM*_L_ > 140nm that cannot be assigned to any particular quantized level were also observed and these were ignored during the screening (designated as ‘U’ in S8B Fig) process. Although pixel intensities vary depending on the number of molecules, excitation intensity distribution, dipole orientation, etc., the maximum quantized discrete intensity value apparently did not exceed beyond 6-fold. This led to conclude that the maximum number of ehSTIM1 dimer units that can be formed within ER-PM junction is indeed a hexamer–a single molecule containing 6 ehSTIM1 dimer units.

Theoretical estimates of the number of emitters can also be obtained by applying statistical models described elsewhere [33] to the intensity distribution. An apparent number of emitters, *N*_app_ defined as the squares of the ratio of mean and standard deviation of the pixel intensities can be a useful metric to assess the relative number of emitters present in z-steps. *N*_app_ depends on the scan speed [33], which was fixed due to the constant pixel dwell time across all the scanned frames. S9A Fig shows a plot of the *N*_app_ characterizing the number of emitters across different z-steps. The average number of emitters within the cluster shown in Fig 4A is predicted to be *Ñ* ~ 4.63 ± 0.36 based on *N*_app_ and this agrees closely with the experimentally determined average number of emitters (*Ñ*_exp_ *=* 4) based on intensity quantization rule (See S9B Fig and Materials and Methods).

**Fig 6:**
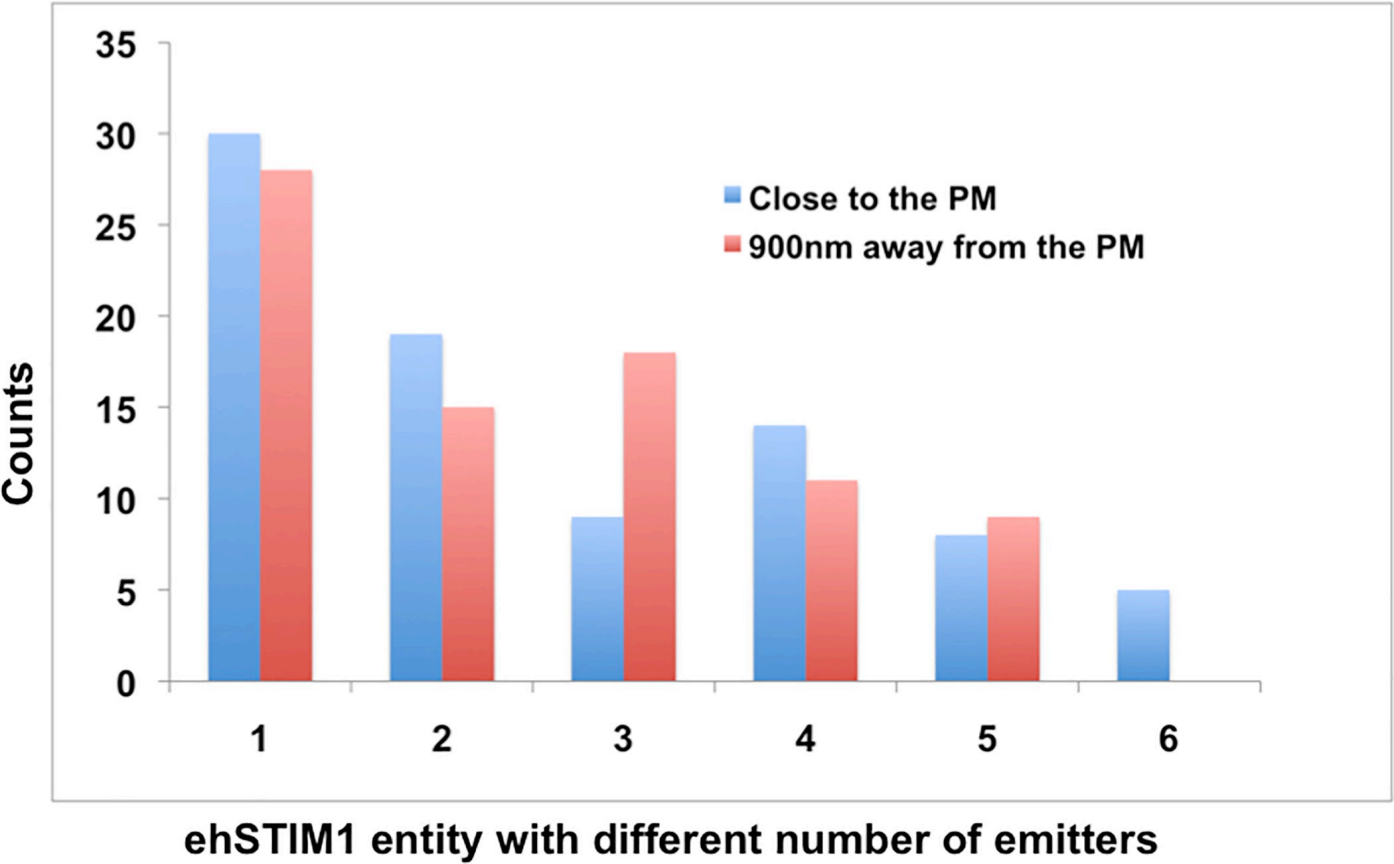
ehSTIM1 entities associated with six emitters are more in the vicinity than away from the PM. Distribution of different number of emitters characterizing co-localization of various ehSTIM1 oligomeric species in the vicinity of the PM and about 900nm away from the PM inside the chosen representative cell. Molecular entities representing colocalization of oligomeric species with six emitters were observed in the vicinity of the PM.

Quantization of discrete intensities of diffraction-limited fluorescent spots can provide new insights into the molecular arrangement of ehSTIM1 oligomers. Fig 6 shows the distribution of a number of emitters in 24 randomly selected clusters at close proximity to and about a ~ 0.9*μ*m away from the PM respectively. A total of 84 and 78 molecular entities with a different number of emitters were identified from 24 randomly chosen clusters screened close to and away from the PM respectively (See S10 Fig for the distribution of the chosen clusters). Molecular entities with a definitive number of emitters were deduced applying quantization rule and the lateral resolvable limit posed by *FWHM*_L_ of respective PSFs within each cluster. It is evident that a diverse set of ehSTIM1 dimer units localize both close to and away from the PM even when ORAI1 is not sufficiently over-expressed. One-way ANOVA (Analysis of variance) statistical hypothesis tests confirmed a statistically significant difference (at *α* = 0.05) in the mean number of emitters per cluster (See S2 and S3 Tables). In particular, ehSTIM1 entities with six emitters have a greater probability of occurrence close to the PM. This is clearly manifested by the increase in their count close to PM compared to that away from it (See Fig 6). However, the number density of the entities with six emitters is smaller than that of others even in the proximity of PM. Furthermore, the mean counts per cluster of any pseudo representation are higher and not equal to the total mean counts per cluster for an entity with six emitters. This is true for both the cases (close to and away from the PM), which, suggests a significant contribution of the true single hexamer in the screened entities. It is not known if hexameric species are formed locally close to the PM because apparently no entities with six emitters were observed in the chosen clusters away from the PM. Also, diffusion of higher oligomeric species of ehSTIM1 to ER-PM junction from the sites of initial activation might take longer than the duration required for puncta formation (a few tens of seconds [10]). Irrespective of whatever is attributed to the formation of hexameric species, several puzzling questions still remain; What is the underlying molecular mechanism that imposes a restriction on the oligomerization states of ehSTIM1 dimers beyond six? If hexameric ehSTIM1 species are produced locally close to PM, can it be attributed to the geometric effects of ER-PM junction or to proteins localized at ER-PM junction or to the PM targeting by phosphoinositides [10, 12] and endogenous ORAI1 [12]? Recently TMEM110 [34] was identified as a modulator of STIM1 conformation at ER-PM junctions for regulating the Ca^2+^ influx. However, its mode of interaction with STIM1 is not understood yet. Detailed super-resolution imaging of ehSTIM1 co-expressed with ORAI1 localized in the ER-PM junctions at varied expression levels will indeed enable answering these questions.

The number of ORAI1 subunits that constitutes a functional channel in cells has been a subject of controversy over the last few years. Electrophysiology recordings from cells expressing engineered ORAI1 concatemers reported conflicting results [35–38]. On the other hand, single molecule fluorescent bleaching [39] and fluorescence calibration [24] approaches predicted the functional channel as a tetramer. My findings here based on screening ehSTIM1 puncta at diffraction-limited resolution led to postulate that ehSTIM1 dimers exist in bundles ranging from 1–6 but with a greater probability of occurrence for hexameric species in the proximity of PM compared to other lateral sections. Given the low number density of ehSTIM1 entities with six emitters close to PM and a 10% probability for an entity to be a true hexamer, it is very tempting to speculate if these correspond to endogenous ORAI1 sites in the islands of PI(4,5)P_2_ given the probable stoichiometries of STIM1-ORAI1 complexes [40–41] and the channel activity configurations [42] reported previously. Since ehSTIM1 alone is over-expressed, it is impossible to confirm from the current set of experiments if endogenous ORAI1 is localized at the sites of hexameric species close to the membrane.

## Summary

Taken together, this work confirms three findings; (i) luminal domains of engineered hSTIM1 monomer units interact triggering ehSTIM1 activation after a drop in Ca^2+^ concentration inside ER lumen, (ii) Activated ehSTIM1 occurs in a diverse set of oligomeric states ranging from 1–6 dimer units with increased probability of occurrence of hexamers near the PM (iii) The number density of hexameric species is lower than that of other species. Multiple ORAI1 gating states defined by the modulation of the binding affinities with ehSTIM1 can influence the channel currents as observed in Orai1 concatemers [42]. Although these Ca^2+^ currents are very small, they are diverse secondary messengers capable of regulating various several cellular functions–cell division, gene expression, apoptosis, etc. Hence, spatial mapping of the distribution of the ehSTIM1 oligomers and quantitating the molecular density of PM targeted species are indispensable. Furthermore, there has been an intrinsic interest in the development of small molecule drugs for modulating the CRAC channel activity but most candidates have very poor selectivity [43]. Enumerating the number density of ehSTIM1 oligomer species in the vicinity of the PM will improve our understanding to engineer efficient modulators of STIM1-ORAI1 coupling machinery. Also, the results presented here demonstrate the potential of live functional imaging to visualize protein-protein interactions at a diffraction-limited resolution in cells. The relevant molecular engineering and the accompanying functional imaging approaches can also be applied for dissecting other signaling pathways in cells.

## Materials and methods

### Molecular cloning of engineered hSTIM1 constructs

The complementary DNA encoding the fusion protein eGFP-hSTIM1 cloned in a mammalian vector pCMV-XL5 used in several earlier experiments [44,5] was obtained from Dr. Patrick Hogan Laboratory (See S1 Fig and S1 Table). Using this plasmid DNA as the starting material, two plasmids—eGFPS1-hSTIM1 and eGFPS2-hSTIM1 encoding complementary eGFP split fragments fused to N-terminal of hSTIM1 were created by deletion mutagenesis reaction. eGFPS1 and eGFPS2 are split pairs expressing 1-157aa and 158-240aa respectively. Quickchange lightning site directed mutagenesis reactions (Catalog #210518, Agilent genomics) were performed following manufacturer’s protocol to create two plasmids encoding eGFPS1-hSTIM1 and eGFPS2-hSTIM1 respectively. Single strand DNA oligomers (5'-ttgatgccgttcttggcgcccgagctgg-3' and 5'-ccagctcgggcgccaagaacggcatcaa-3'; 5'-atggccgacaagcaggccaactctgaggag-3' and 5'-ctcctcagagttggcctgcttgtcggccat-3') were used as primers for sense and anti-sense strands of the cDNA to create eGFPS1-hSTIM1 and eGFPS2-hSTIM1 plasmids respectively. Five randomly picked bacterial colonies were separately inoculated into 3mL LB media containing 1*μ*L/mL of 100mg/mL Ampicllin stock were cultured for 12hrs at 37°C at 225 rpm. Plasmid DNAs purified from monoclonal colonies were sequenced to identify positive clones. Samples of positive clones that yielded high quality reads (Q>45) were further sequenced to ensure 100% match for nucleobases encoding the cDNA.

### Reagents

All reagents unless otherwise stated were purchased from Sigma Aldrich (highest grade).

### In vitro expression of various hSTIM1 constructs in mammalian cells

The cDNAs encoding eGFPS1-hSTIM1, eGFPS2-hSTIM1, and eGFP-STIM1 were cloned in the mammalian vector, pCMV-XL5. HeLa cells (from ATCC) were then chemically transfected by plasmid DNAs for live fluorescent imaging. The experimental protocol involves expanding a frozen stock of low passage (<15) HeLa cells in a freshly prepared growth media containing Dulbecco’s Modified Eagle’s Medium (DMEM, #11995–040; Gibco), 10% heat-inactivated fetal bovine serum, 100u/mL streptomycin, 2mm L-glutamine, nonessential amino acids, sodium pyruvate, and 10mm HEPES. The cells were cultured in an incubator containing 10%CO_2_, and 87%RH maintained at 37°C. The cells were allowed to recover over two passages by splitting at 1:10 ratio each time after they reach 80% confluent on a 10 cm tissue culture dish. Each splitting process involves aspirating out the media, followed by a gentle wash using phosphate buffered solution (Affymetrix USB), treatment with 1mL of Trypsin-EDTA spread over the plate and incubation at 37°C for 1 min. The Trypsin activity was stopped by adding 9mL of growth media into the plate thereby causing the cells to suspend in the solution. The suspended cells were then subjected to differential centrifugation (1000g for 5 min) to pellet them at the bottom of a 14mL tube. This is followed by another round of wash using growth media to reduce the residual Trypsin if any in the pellets. The cells were then plated onto a 6-well plate at a seeding density of 5x10^5^ cells per well. After 24hrs, the cells were transfected with 1*μ*g of plasmid using commercial Lipofectamine 2000 reagent (Invitrogen) following the manufacturer’s protocol. After 7hrs, a fresh media replaced the media containing the transfection mix. For co-expression of eGFPS1-hSTIM1 and eGFSPS2-hSTIM1 500ng of each plasmid was used for preparing the transfection mix.

Meanwhile, a 12-well plate with an 18mm diameter glass coverslip (Catalog #72229–01, Electron Microsccopy Sciences) in each well was washed with 500*μ*L of 1m HCl for 30min followed by ample washing (thrice with 1mL each time) with DI water. The washed coverslips were subsequently treated with 500*μ*L of 100% ethanol for 30mins followed by ample DI water wash step. The washed coverslip in each 12 well plate was UV-sterilized for 12hrs. After 24hrs post-transfection, the cells were gently washed by 500*μ*L of 1x Phosphate Buffered Solution (PBS) and treated with 500*μ*L of trypsin-EDTA for 1 min to dislodge the cells from the attached plate. Adding 5mL of media directly into the well stopped the enzymatic activity of trypsin. The dislodged cells were then briefly centrifuged (1000 rpm for 5min) and washed with 5mL media once to bring down the concentration of residual active trypsin if any to a negligible amount. The cells were then re-suspended in 5mL of media and 1mL was added into each well of 12-well plate containing UV sterilized coverslip. After 48hours post transfection, Thapsigargin (TG, from Molecular probes) was added into two wells to a final concentration of 1*μ*m and the plate was kept inside the incubator for 20min. Each coverslip was then gently washed with PBS and mounted directly onto a well containing mammalian Ringer’s solution (2.2mm CaCl_2_, 5.6mm KCl, 154mm NaCl, 2.4mm NaHCO_3_, 2mm Tris-HCl, PH7.4). For coverslips treated with TG, the mounting well solution was spiked with TG to a final concentration of 1*μ*m. The wells were created on a glass slide (76mm × 25mm × 1mm) by affixing a doubly adherent secure seal spacer (13mm diameter, Electron Microscopy Sciences) and adding 80*μ*L of mounting well solution. Coverslip was placed inverted on a sticky spacer so that the cells are in contact with mounting well solution. The sealing pad was then placed over the coverslip and gently pressed on the edges to securely seal underneath the well.

Achieving nominal protein expression for the two complementary ehSTIM1 interacting partners is the key for diffraction-limited imaging. The key to achieve diffraction-limited imaging is the finite efficiency in several steps leading to ehSTIM1 oligomerization—sharing of the transcription factors that expresses the complementary ehSTIM1 molecules, dimerization of complementary ehSTIM1 at the resting state, assembly of complementary eGFP domains upon drop in ER Ca^2+^ concentration and maturation of a self-assembled eGFP to a functionally fluorescent domain. These inefficient steps limit the number of activated ehSTIM1 that can actually fluoresce at a diffraction-limited resolution in cells. Perhaps it is possible some eGFP split pairs did not mature completely after luminal domain association and this could not be accurately accounted for in this assay.

### Live imaging of ehSTIM1 using confocal microscopy

Confocal micrographs were obtained using Olympus Fluoview FV10i inverted microscope equipped with a motorized stage, focus control and scanner unit for simultaneous laser stimulation. In-plane phase contrast and fluorescent signals of a live single cell attached to a coverslip were imaged at sub-cellular resolution in different z-stacks. The imaging protocol involves carefully cleaning the exposed surface of a coverslip with lens cleaning tissue soaked in commercial Windex^TM^ solution. After allowing the coverslip to air dry for a couple of minutes, the glass slide was mounted gently on a suitable fixture that sits on the motorized stage. Two diode laser lines at excitation wavelengths of 473nm (15mW) and 559nm (18mW) were chosen to image the eGFP (em: 490-540nm) and RFP (em: 570-620nm) signals from the cells respectively. eGFP signal is the readout for engineered hSTIM1 molecules and RFP is the readout for ER lumen (ER-Red mammalian vector that express RFP-Calreticulin from Thermo Fisher Scientific). All laser lines were automatically chosen to deliver between 2–3% of the available maximum power at 100%. Laser inputs exceeding 12% resulted in fluorescent bleaching of eGFP molecules. The detector sensitivities i.e. analog photomultiplier tube (PMT) voltages for fluorescent and phase contrast imaging were set to 40% (500mV) and 35% (334mV) respectively. The PMT settings sensitize detectors to detect green photons even for nominal expression levels of eGFP-hSTIM1. An initial scan covering a large area around the centre of the coverslip was performed to identify a suitable field of view. The scan speed was set to 4x and the imaging option was set to balance between speed and quality. Variable confocal aperture with the available range (pinhole diameter = 50–800 *μ*m diameter) was set to 5x (pinhole diameter = 105*μ*m). Variation in the expression levels of different hSTIM1 constructs in cells, effect of background and cell debris artefacts were visually assessed and their respective locations were registered in the chosen field of view using 10x/0.4 air objective. Healthy and viable cells expressing hSTIM1 constructs at nominal levels (LUT value of ~2000 with 12 bits per pixel) were chosen to image under 60x/1.35 Oil objective (UPLSA60xO) magnified by 3.4x optical zoom from the registered locations. Single cell images captured under these conditions were resolved by 1024 x 1024 pixels at a pixel size of ~60nm. For imaging z-stacks of a cell, the slider was allowed to travel between the footprint and top of the cell. The location of the footprint and the top of a cell were determined by comparing the trends of actual fluorescent intensity against the background for a smallest resolvable slider motion in the z-direction. The number of z-steps varies depending on the total height of a cell (~ 8–9*μ*m) proportionate to the step size between ~300nm. Cells adhered to coverslips were treated with TG for 30mins before imaging. This duration is sufficient for the self-assembly of complementary eGFP split pairs to form a fully matured fluorescent eGFP molecule [19].

Single cell images were captured at a pixel resolution of 1024x1024 at a pixel size of ~ 60nm. Lateral and axial resolution quantified by full-width half maximum (*FWHM*) of a confocal microscope can be obtained as a function of numerical aperture, *NA*, the refractive index of the medium, *n* and the wavelength of the light, *λ* [30] as

$$FWHM_{L}=\frac{0.37\lambda }{NA};\;FWHM_{A}=\frac{0.64\lambda }{n-\sqrt{n^{2}-NA^{2}}}$$ (1)

where *FWHM*_L_ and *FWHM*_A_ are full width at half maximum in the lateral and axial directions respectively. Substituting *λ* = 510 nm (spectral wavelength for the maximum emission of an eGFP molecule), NA = 1.35 (for a UPLSA60xO objective), *n* ~1.5 in (1) gives *FWHM*_L_ ~140nm and *FWHM*_A_ ~386nm. Corresponding values achievable from a wide field microscopy are approximately greater by √2 fold [30] suggesting its limitation on precise localization of single molecules. For a square pixel of ~ 60nm size and an axial resolution limited by *FWHM*_A_ ~ 386nm, a smallest possible observation volume can be approximated by a cylinder of radius, *r*_c_~ 140nm and axis length, 2.*l*_c_~ 386nm. Using the mean diffusivity of activated hSTIM1 ensemble obtained from FRAP (Fluorescent recovery after photobleaching) experiments (*D*~ 0.08 μm^2^/s [12]), the time taken for an activated ehSTIM1 inside an approximated cylindrical voxel to laterally diffuse away would be *τ*_diff_ = r_c_^2^/4*D* ~ 61ms. This duration is four orders of magnitude greater than the dwell time of PMT (2*μ*s/pixel) for detecting green photons emitted from a voxel.

### Image processing methods

Single cell confocal fluorescent micrographs were processed using ImageJ software (www.imagej.nih.gov/ij/). Z-steps captured were projected and then background subtracted (using a rolling ball radius of 50 pixels). The micrographs were despeckled to remove salt and pepper noise if any, and all the bright outliers were removed (radius = 2 pixels, threshold = 50 pixels) to remove hotspots.

The micrographs were converted to 8-bit color and average pixel intensity within the field of view was evaluated (See S5A Fig). The approach for computing Shannon entropy of the 8-bit image was already discussed in detail elsewhere [25]. MATLAB in-built *entropy* routine was used to evaluate the average entropy of each 8-bit image. S5B Fig shows the effective number of gray levels, I_eff_ = 2^Hm^ encoding the ehSTIM1 oligomerization using mean Shannon values. It is evident from S5A and S5B Fig that depletion of Ca^2+^ causes luminal domain association of ehSTIM1 in cells. It is obvious from S5C Fig that average eGFP fluorescence correlates with the entropy for all stacks from the footprint to the top surface of the cell.

Diffraction-limited molecular imaging of ehSTIM1 molecules forming puncta was spatially resolved and filtered using convolution filter to the image stacks. A 5x5 edge detection kernel was applied so that a normalized weighted average of 24 neighbors of each pixel element is represented on the image. S6 Fig shows a 3D surface plot of diffraction limited intensity contours of a particular z-stack shown in Fig 4A. Point-spread functions representing different number of emitters were spatially resolved to enumerate possible oligomeric combinations.

Plausible oligomeric combinations of ehSTIM1 molecular entities separated at least by *FWHM*_L_ were enumerated by discrete quantization of peak intensities that scales almost linearly with the number of emitters as shown in Fig 4B. A sum of four Gaussian functions described by means, *μ*_n_ and the standard deviations, σ_n_ that best fit the multiple point-spread functions was obtained by nonlinear curve-fitting algorithm using MATLAB. An equation of the form

$$y=\sum_{n=1}^{4}a_{n}.e^{-{\left(\frac{x-\mu_{n}}{\sigma_{n}}\right)}^{2}}$$ (2)

was obtained where a_n,_ *μ*_n_ and *σ*_n_ are constants associated with the four peaks denoted by, *n* = 1,2,3 and 4. The independent variable, *x* and the dependent variable, *y* denote the pixel location and the intensity values. The constants were estimated using the in-built MATLAB routine, *cftool*. Non-linear least square method applying Trust-region algorithm at a function tolerance value of 10^−6^ for 1000 iterations was chosen to solve for the constants. Since a number of solutions are possible to achieve a good fit, the most appropriate fit was obtained by constraining the allowable range for the constants so that the peaks are located within the required intervals. An optimal solution (*a*_*1*_ = 39.52, *μ*_*1*_ = 5.88, *σ*_*1*_ = 1.04, *a*_*2*_ = 65.27, *μ*_*2*_ = 7.88, *σ*_*2*_ = 2.69, *a*_*3*_ = 17.28, *μ*_*3*_ = 9.95, *σ*_*3*_ = 0.24, *a*_*4*_ = 12.85, *μ*_*4*_ = 17.03, *σ*_*4*_ = 0.91) with an R^2^ ~ 99% suggests that the curve fit agrees with the raw data excellently.

Theoretical estimates of the number of emitters were predicted using the statistical approaches described elsewhere [33]. An apparent number of emitters, *N*_app_ = (*î*_p_)^2^/***Var*** for each frame was evaluated by using the mean, *î*_p_ and variance, ***Var*** of the pixel intensities. S9A Fig shows a plot of *N*_app_ for different frames included in the montage plot S7 Fig. Average number of emitters, *Ñ* was determined using the relation

$$\tilde{N}=\frac{\delta .\ln\left(2.l_{c}/r_{c}\right)}{\left(\theta /\tau_{\mathrm{diff}}\right)+{\left(\theta /\tau_{\mathrm{scan}}\right)}^{2}}$$ (3)

where *δ* is the mean of the best fit Poisson distribution describing the image histogram, (2.*l*_c_/*r*_c_) ~ 5.5 is the geometric factor of the observation volume approximated by a cylinder of radius, *r*_c_ and half-axis length, *l*_c_; θ is the pixel dwell time, *τ*_diff_ = r_c_^2^/4*D* is the lateral diffusion time of the emitter with diffusivity, *D* ~ 0.05 *μ*m^2^/s and *τ*_scan_ = 2*r*_c_.*θ* /*p*_w_ is the scan time of a pixel with pixel width, *p*_w_ ~ 61 nm. S9B Fig represents Poisson modeling of the pixel intensity distribution of the emitters shown in Fig 4A. Using the maximum likelihood estimation routine, *mle* in the MATLAB software, a mean value, *δ*_mle_ *~* 0.0039 per pixel value was estimated. A total of 33 allowable gray values have an illumination probability of ~0.0035 which is approximately equal to *δ*_mle_. These gray values exceed a pixel intensity value of 17, which is higher than the background intensity value (< 6) and together they represent the emitters localized within the chosen window. This can be inferred indirectly by evaluating the average number of emitters using Eq (3) assuming *δ =* 33 × *δ*_mle_ = 0.1287. Theoretically estimated number of emitters, *Ñ* ~ 4.63 ± 0.36 obtained using (3) agrees closely with the experimentally determined value, *Ñ*_exp_ = (6+5+4+1)/4 = 4

### Statistical analysis

A Gaussian model was used to curve fit the point-spread function of the spatially localized emitters. Raw images recorded were processed as described in the earlier section. Several single-cell images from three unblinded biological repeats were recorded of which three representative cells were shown. Within a chosen cell, ehSTIM1 clusters at 24 random spatial locations were considered at frames 5 and 8 for screening the oligomeric species localized within the respective focal planes. The number of emitters representing possible combinations of different oligomeric species for the two different cases—at the proximity of PM and about 0.9 *μ*m away from the PM were determined based on the criteria set by the intensity quantization rule and *FWHM*_L_ thresholds on the PSFs. One-way ANOVA statistical hypothesis test was conducted to ensure significant difference exists between the mean counts of different number of emitters within the randomly chosen clusters (See S1 and S2 Tables). The null hypothesis, *H*_0_ and the alternative hypothesis, *H*_1_ state the mean counts of all emitters are equal and unequal respectively. The computed test statistic, F (3.875 and 2.865 for the two cases) is higher than the threshold value (~2.3) at 95% confidence level thereby suggesting that *H*_0_ has to be rejected for both the cases considered.

## Supporting information

S1 Fig pCMV-XL5 mammalian expression vector encoding eGFP-hSTIM1. Molecular cloning was performed in bacteria before expressing the engineered hSTIM1 proteins in mammalian cells. S1 Table lists the genetic and the amino acid sequences of the eGFP-hSTIM1.
(PDF)

S2 Fig Representative ehSTIM1 puncta near the PM in a TG treated cell from one of the biological replicates. Two cells (indicated in the yellow font as 1 and 2) within the chosen field of view show engineered hSTIM1 with eGFP split pairs self-assembled after depletion of ER Ca^2+^ level. Several cells co-expressing eGFPS1-hSTIM1 and eGFPS2-hSTIM1 exhibited this property. Most cells show a very low fluorescence after TG treatment suggesting engineered hSTIM1 with one of the split pairs is not sufficiently expressed in those cells. A total of three replicates were performed and a similar behavior was observed in all the replicates.
(PDF)

S3 Fig Co-expression of eGFP-hSTIM1 and RFP-Calreticulin in HeLa cells under untreated and TG treated conditions. Activated eGFP-STIM1 forms puncta in cells treated with Thapsigargin. However, eGFP-hSTIM1 molecular densities at these expression levels are too high to be resolved for the diffraction-limited imaging. The last column shows superimposition of fluorescent and phase contrast images. The scale bar length is 5*μ*m.
(PDF)

S4 Fig Evidence placed confirming increase in the number of illuminated pixels on a TG-treated cell compared to an untreated cell. (a) Image histogram illustrating that maximum pixel value of the Green channel does not exceed 1 (Mean ~ 0.003) for a representative untreated cell shown in Fig 2C. (b) In contrast, TG treated representative cell shown in Fig 2D has a diverse illuminated pixel values (Mean~10.583 and Max = 255). This confirms the luminal domain association of ehSTIM1 proteins after a drop in Ca^2+^ concentration in cells.
(PDF)

S5 Fig Fluorescent signal is a proxy for luminal domain association of eGFPS1-hSTIM1 and eGFPS2-hSTIM1 in TG treated cells. (a) mean fluorescent intensity of a representative cell expressing engineered hSTIM1 proteins under different conditions and (b) Effective number of pixel gray values, I_eff_ calculated using the Shannon entropy, (c) Mean fluorescent intensity and I_eff_ correlates positively at various Z-steps through a cell.
(PDF)

S6 Fig Localization density of a population of ehSTIM1 oligomers after TG treatment. Diffraction-limited 3D intensity contour clearly discerns different number of emitters in the focal plane for the chosen z-step.
(PDF)

S7 Fig A montage of ehSTIM1 fluorescent puncta at a diffraction-limited resolution for a TG treated cell. Images were captured for an incremental z-step ~ 300nm. Detecting PM localized single molecules if any is possible only at the extreme Z-locations (foot print or at the top surface of a cell).
(PDF)

S8 Fig Illuminated pixels representing colocalization of ehSTIM1 oligomers at various z-steps. (a) Several windows containing ehSTM1 clusters were considered for intensity quantization and a representative from each frame is shown (b) Corresponding intensity traces of the illuminated pixels from the representative windows. The abscissa and ordinate of the plots represent pixel counts and intensity values (in arbitrary units) respectively. Multiple discretely quantized intensity levels representing different number of emitters localised within the chosen cluster are shown. The localization of peaks observed in frames 6, 8 and 9 cannot be assigned to any particular quantized intensity levels and hence denoted as ‘U’.
(PDF)

S9 Fig Estimation of apparent number of molecules and the average probability of illumination. (a) An apparent number of emitters in each frame were calculated using the statistical approach described earlier [33]. (b) Intensity histogram associated with the illuminated pixels shown in Fig 4A. Assuming the intensity histogram follows a Poisson distribution, a mean value, δ_mle_ ~ 0.0039 per digitized intensity value was obtained applying maximum likelihood estimation.
(PDF)

S10 Fig Randomly selected ehSTIM1 clusters considered for ANOVA analysis. Intensity quantization rule was applied to ehSTIM1 clusters within each yellow window to quantitate oligomeric species associated with different number of emitters.
(PDF)

S1 Table DNA and amino acid sequences of ehSTIM1. *eGFP-hSTIM1* was cloned in the pCMV-XL5 vector whose plasmid map was shown in S1 Fig.
(PDF)

S2 Table Statistical analysis of the distribution of emitters localized in the vicinity of the PM. A one-way ANOVA was performed using the raw data shown in Fig 6.
(PDF)

S3 Table Statistical analysis of the distribution of emitters localized ~ 900nm away from the PM. A one-way ANOVA was performed using the raw data shown in Fig 6.
(PDF)
