# Supplementary figures and images for: Detecting In-Situ oligomerization of engineered STIM1 proteins by diffraction-limited optical imaging
Source: PLoS One. 2019 Mar 25;14(3):e0213655. doi: 10.1371/journal.pone.0213655 (PMC6433367; doi:10.1371/journal.pone.0213655)

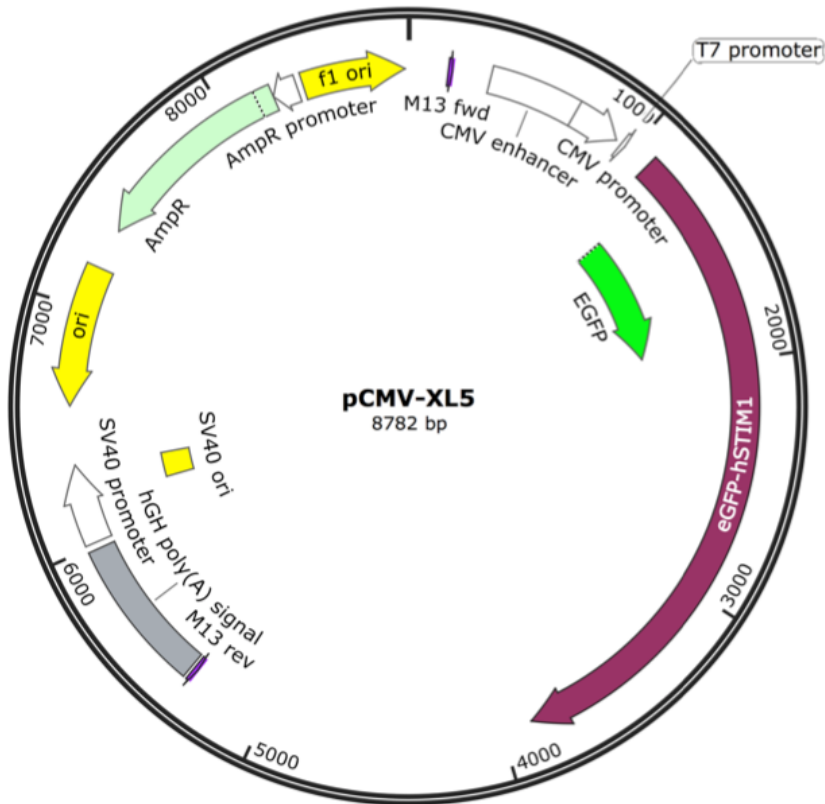

Supplement: S1 Fig — Molecular cloning was performed in bacteria before expressing the engineered hSTIM1 proteins in mammalian cells. S1 Table lists the genetic and the amino acid sequences of the eGFP-hSTIM1. (PDF) [file pone.0213655.s001.pdf]

**Fluorescence**

**Phase contrast**

**Merge**

**Experiment 2**

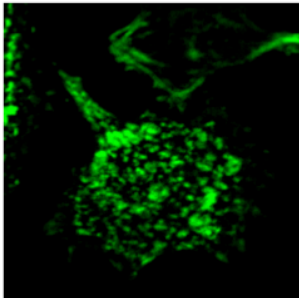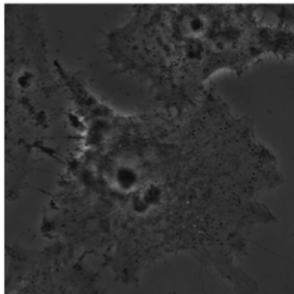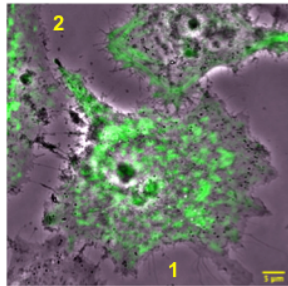

Supplement: S2 Fig — Two cells (indicated in the yellow font as 1 and 2) within the chosen field of view show engineered hSTIM1 with eGFP split pairs self-assembled after depletion of ER Ca2+ level. Several cells co-expressing eGFPS1-hSTIM1 and eGFPS2-hSTIM1 exhibited this property. Most cells show a very low fluorescence after TG treatment suggesting engineered hSTIM1 with one of the split pairs is not sufficiently expressed in those cells. A total of three replicates were performed and a similar behavior was observed in all the replicates. (PDF) [file pone.0213655.s002.pdf]

Untreated

eGFP-hSTIM1

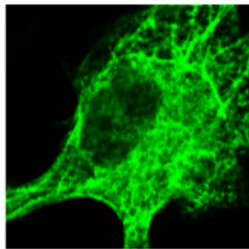

RFP-Calreticulin

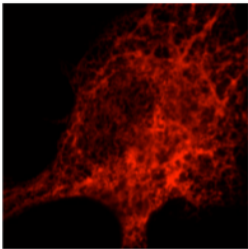

Phase contrast

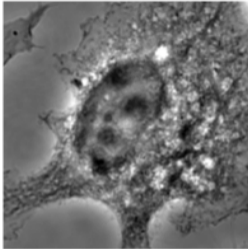

Merge

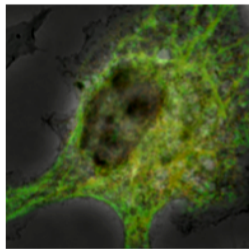

TG-treated

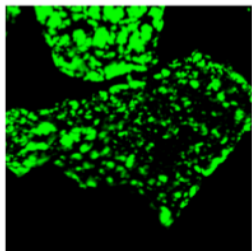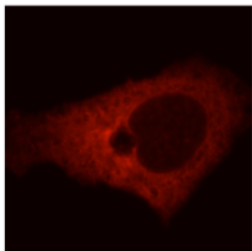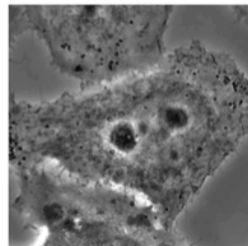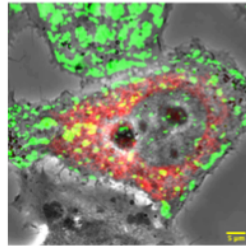

Supplement: S3 Fig — Activated eGFP-STIM1 forms puncta in cells treated with Thapsigargin. However, eGFP-hSTIM1 molecular densities at these expression levels are too high to be resolved for the diffraction-limited imaging. The last column shows superimposition of fluorescent and phase contrast images. The scale bar length is 5μm. (PDF) [file pone.0213655.s003.pdf]

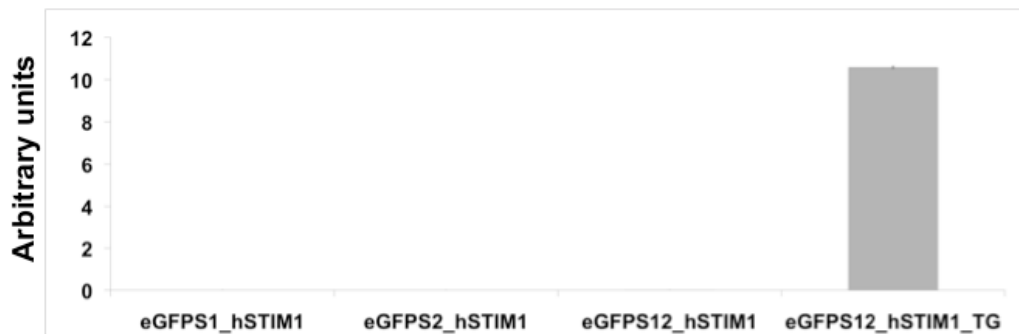

(a)

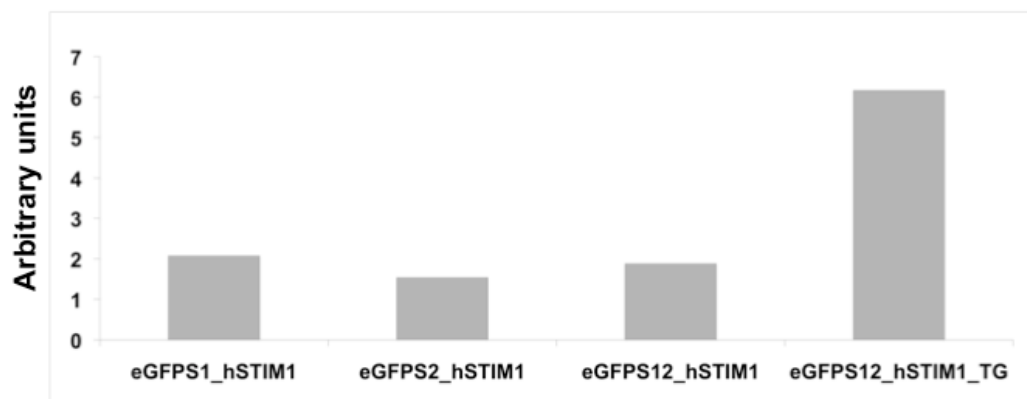

(b)

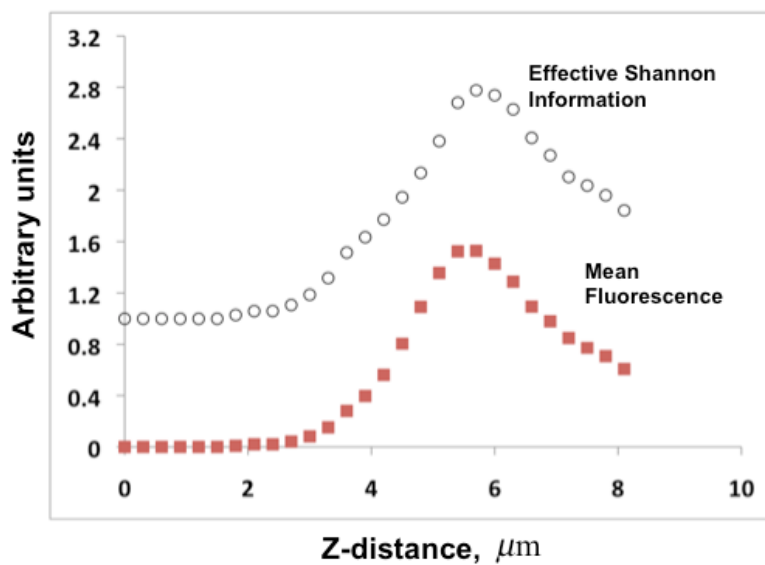

(c)

Supplement: S5 Fig — (a) mean fluorescent intensity of a representative cell expressing engineered hSTIM1 proteins under different conditions and (b) Effective number of pixel gray values, Ieff calculated using the Shannon entropy, (c) Mean fluorescent intensity and Ieff correlates positively at various Z-steps through a cell. (PDF) [file pone.0213655.s005.pdf]

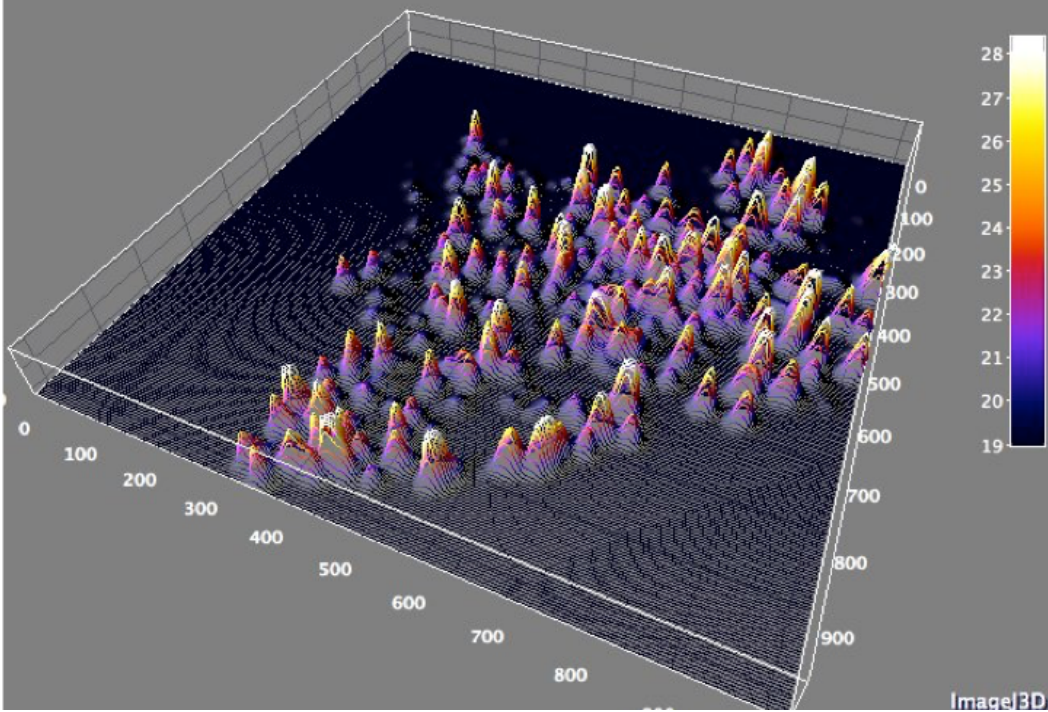

Supplement: S6 Fig — Diffraction-limited 3D intensity contour clearly discerns different number of emitters in the focal plane for the chosen z-step. (PDF) [file pone.0213655.s006.pdf]

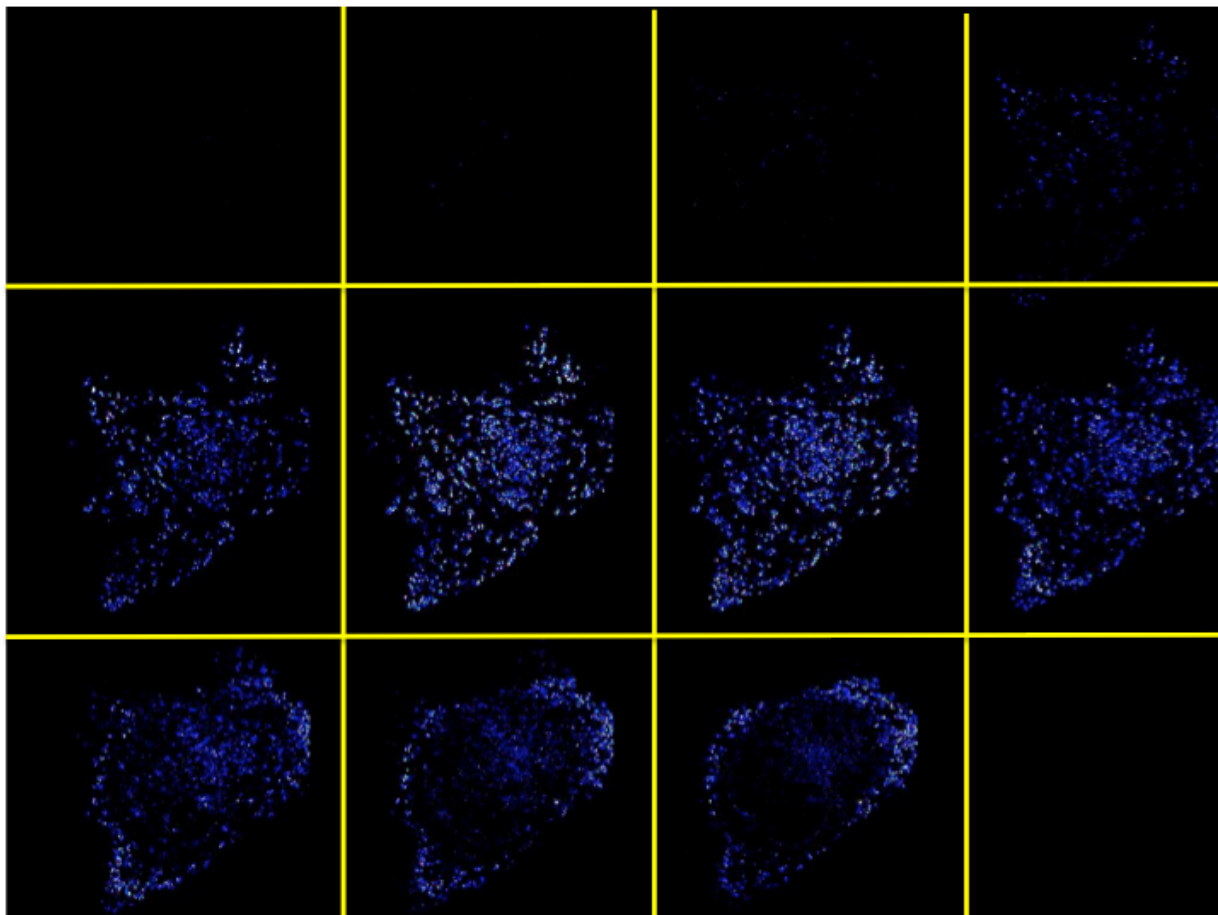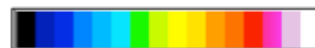

Min

Max

Supplement: S7 Fig — Images were captured for an incremental z-step ~ 300nm. Detecting PM localized single molecules if any is possible only at the extreme Z-locations (foot print or at the top surface of a cell). (PDF) [file pone.0213655.s007.pdf]

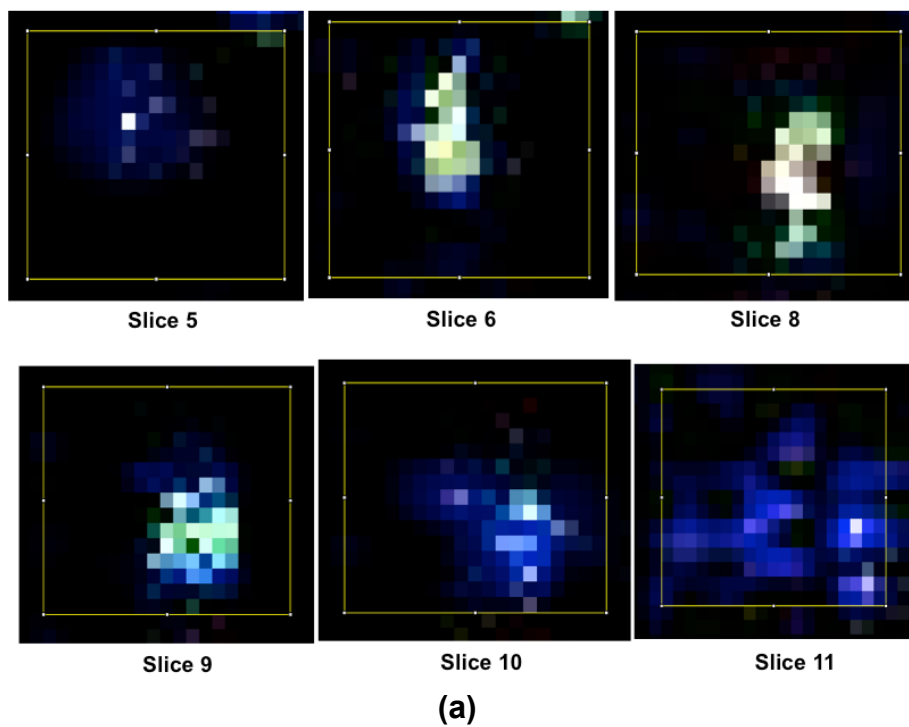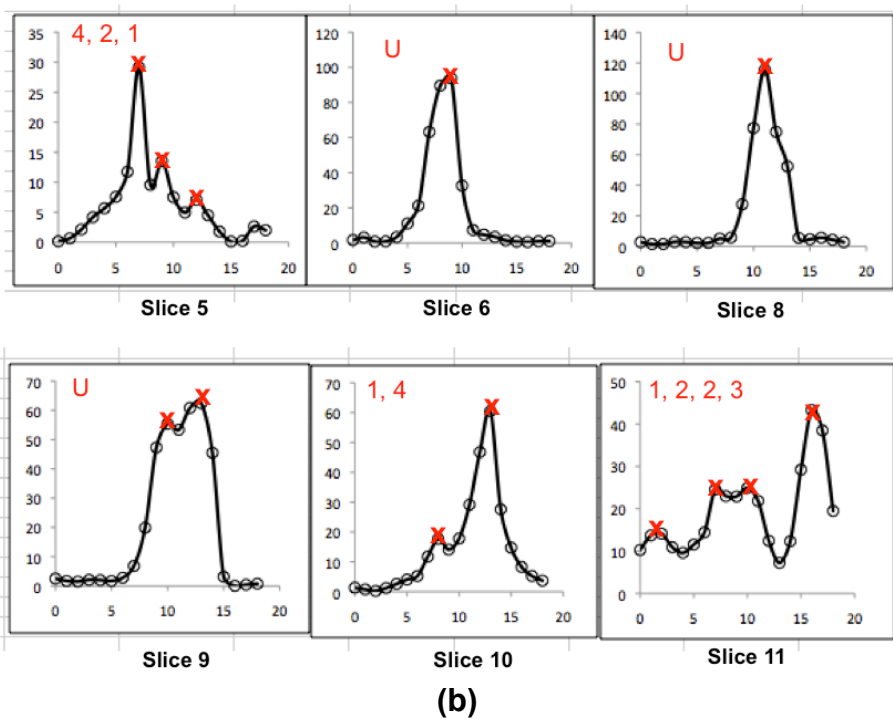

Supplement: S8 Fig — (a) Several windows containing ehSTM1 clusters were considered for intensity quantization and a representative from each frame is shown (b) Corresponding intensity traces of the illuminated pixels from the representative windows. The abscissa and ordinate of the plots represent pixel counts and intensity values (in arbitrary units) respectively. Multiple discretely quantized intensity levels representing different number of emitters localised within the chosen cluster are shown. The localization of peaks observed in frames 6, 8 and 9 cannot be assigned to any particular quantized intensity levels and hence denoted as ‘U’. (PDF) [file pone.0213655.s008.pdf]

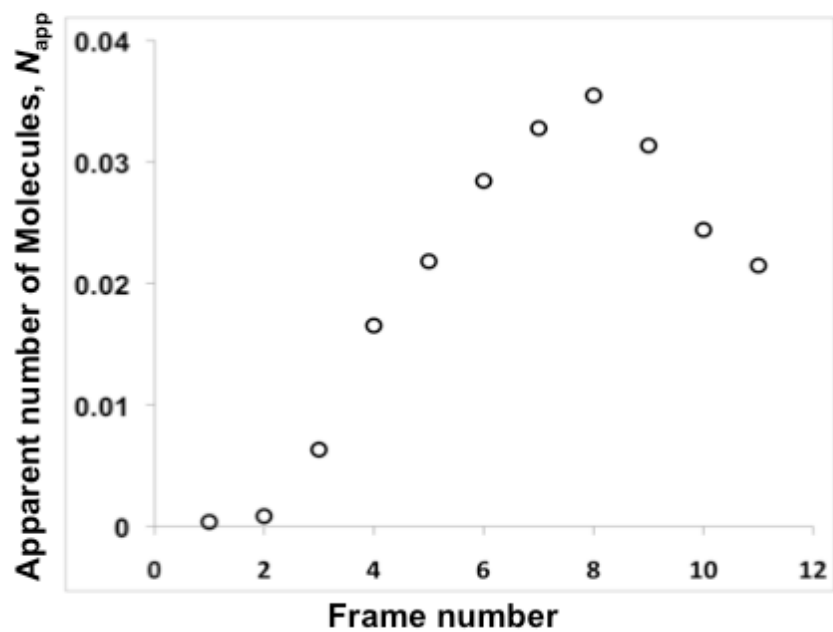

(a)

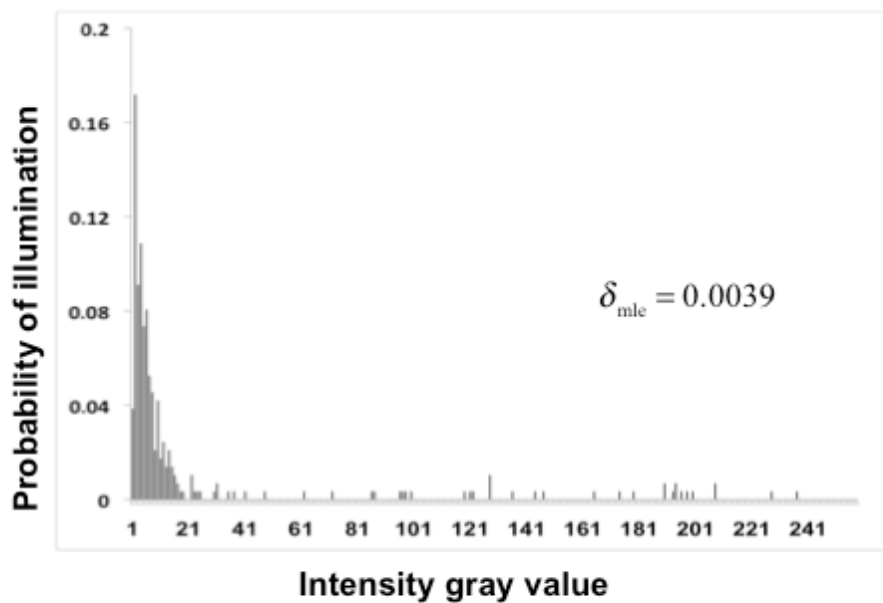

(b)

Supplement: S9 Fig — (a) An apparent number of emitters in each frame were calculated using the statistical approach described earlier [33]. (b) Intensity histogram associated with the illuminated pixels shown in Fig 4A. Assuming the intensity histogram follows a Poisson distribution, a mean value, δmle ~ 0.0039 per digitized intensity value was obtained applying maximum likelihood estimation. (PDF) [file pone.0213655.s009.pdf]

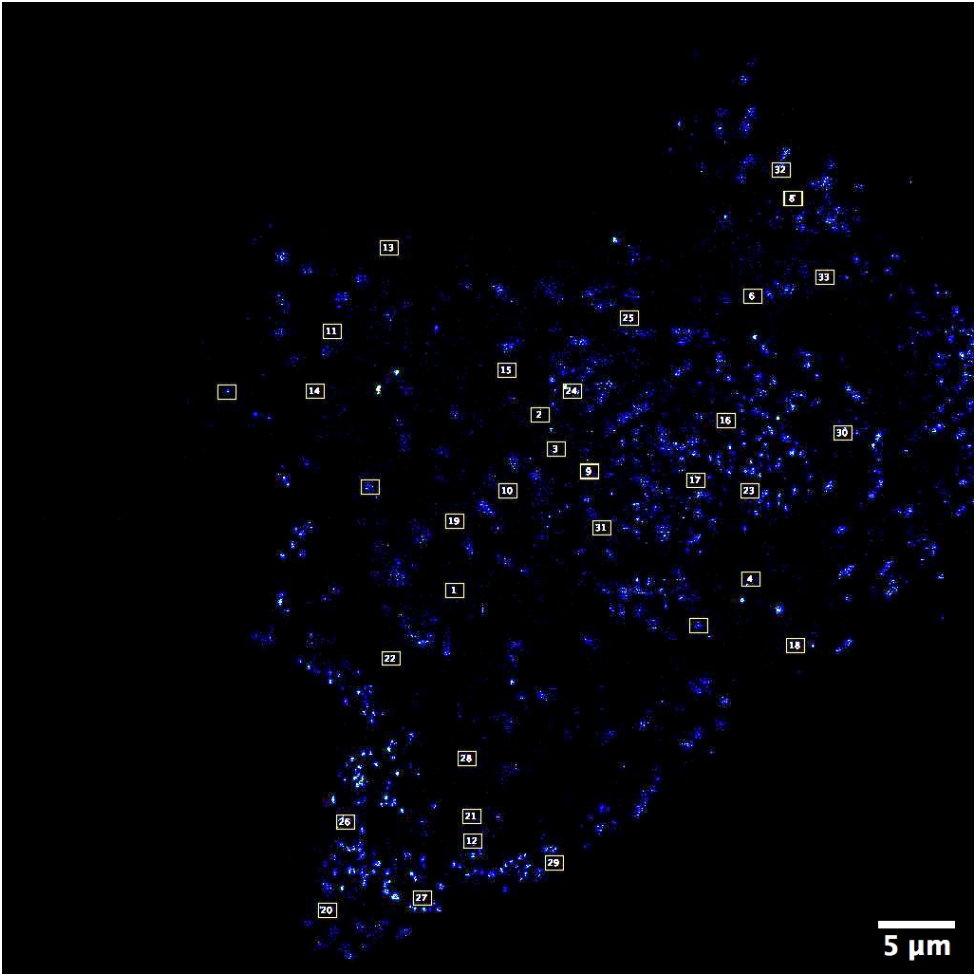

5  $\mu$ m

Supplement: S10 Fig — Intensity quantization rule was applied to ehSTIM1 clusters within each yellow window to quantitate oligomeric species associated with different number of emitters. (PDF) [file pone.0213655.s010.pdf]
