# Supplementary material for: Detecting In-Situ oligomerization of engineered STIM1 proteins by diffraction-limited optical imaging
Source: PLoS One. 2019 Mar 25;14(3):e0213655. doi: 10.1371/journal.pone.0213655 (PMC6433367; doi:10.1371/journal.pone.0213655)
Supplement: S4 Fig — (a) Image histogram illustrating that maximum pixel value of the Green channel does not exceed 1 (Mean ~ 0.003) for a representative untreated cell shown in Fig 2C. (b) In contrast, TG treated representative cell shown in Fig 2D has a diverse illuminated pixel values (Mean~10.583 and Max = 255). This confirms the luminal domain association of ehSTIM1 proteins after a drop in Ca2+ concentration in cells. (PDF) [file pone.0213655.s004.pdf]

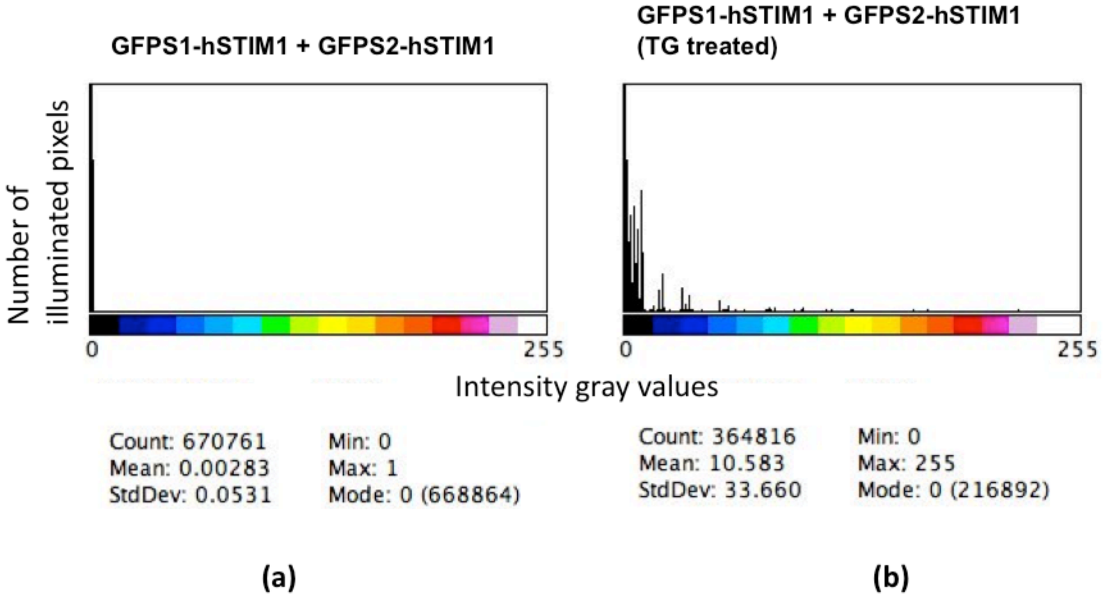
GFPS1-hSTIM1 + GFPS2-hSTIM1
GFPS1-hSTIM1 + GFPS2-hSTIM1
(TG treated)
Number of illuminated pixels
0
255
0
255
Intensity gray values
Count: 670761
Mean: 0.00283
StdDev: 0.0531
Min: 0
Max: 1
Mode: 0 (668864)
Count: 364816
Mean: 10.583
StdDev: 33.660
Min: 0
Max: 255
Mode: 0 (216892)
(a)
(b)
