# Supplementary material for: Detecting In-Situ oligomerization of engineered STIM1 proteins by diffraction-limited optical imaging
Source: PLoS One. 2019 Mar 25;14(3):e0213655. doi: 10.1371/journal.pone.0213655 (PMC6433367; doi:10.1371/journal.pone.0213655)
Supplement: S1 Table — eGFP-hSTIM1 was cloned in the pCMV-XL5 vector whose plasmid map was shown in S1 Fig. (PDF) [file pone.0213655.s011.pdf]

GAGACCGTCGGCTGCACTCCCGGGCTCCTGGCTTTGCCTCTGGGATCCCGAGGTGTCCACATCAGACGCATGTTGACTGAGACCTAGAGTCATGGATGTA 1100
CTCTGGCAGCCGACGTGAGGGCCCGAGGACCGAAACGGAGACCCTAGGGCTCCACAGGTGTAGTCTGCGTACAACTGACTCTGGATCTCAGTACCTACAT

1
M D V
eGFP-hSTIM1

TGCGTCCGTCTTGCCCTGTGGCTCCTCTGGGGACTCCTCCTGCACCAGGGCCAGAGCCTCAGCCATAGTCACAGTGAGAAGGCGACAGGAACCAGCTCGG 1200
ACGCAGGCAGAACGGGACACCGAGGAGACCCCTGAGGAGGACGTGGTCCCGGTCTCGGAGTCGGTATCAGTGTCACTCTTCCGCTGTCCTTGGTCGAGCC

5 10 15 20 25 30 35
C V R L A L W L L W G L L L H Q G Q S L S H S H S E K A T G T S S
eGFP-hSTIM1

GCGCCGTGAGCAAGGGCGAGGAGCTGTTCACCGGGGTGGTGCCCATCCTGGTCGAGCTGGACGGCGACGTAAACGGCCACAAGTTCAGCGTGTCCGGCGA 1300
CGCGGCACTCGTTCCCGCTCCTCGACAAGTGGCCCCACCACGGGTAGGACCAGCTCGACCTGCCGCTGCATTTGCCGGTGTTCAAGTCGCACAGGCCGCT

40 45 50 55 60 65 70
G A V S K G E E L F T G V V P I L V E L D G D V N G H K F S V S G E
eGFP-hSTIM1
1a
EGFP

GGGCGAGGGCGATGCCACCTACGGCAAGCTGACCCTGAAGTTCATCTGCACCACCGGCAAGCTGCCCGTGCCCTGGCCCACCCTCGTGACCACCCTGACC 1400
CCCGCTCCCGCTACGGTGGATGCCGTTCGACTGGGACTTCAAGTAGACGTGGTGGCCGTTCGACGGGCACGGGACCGGGTGGGAGCACTGGTGGGACTGG

75 80 85 90 95 100
G E G D A T Y G K L T L K F I C T T G K L P V P W P T L V T T L T
eGFP-hSTIM1
EGFP

TACGGCGTGCAGTGCTTCAGCCGCTACCCCGACCACATGAAGCAGCACGACTTCTTCAAGTCCGCCATGCCCGAAGGCTACGTCCAGGAGCGCACCATCT 1500
ATGCCGCACGTCACGAAGTCGGCGATGGGGCTGGTGTACTTCGTCGTGCTGAAGAAGTTCAGGCGGTACGGGCTTCCGATGCAGGTCCTCGCGTGGTAGA

105 110 115 120 125 130 135
Y G V Q C F S R Y P D H M K Q H D F F K S A M P E G Y V Q E R T I
eGFP-hSTIM1
EGFP

TCTTCAAGGACGACGGCAACTACAAGACCCGCGCCGAGGTGAAGTTCGAGGGCGACACCCTGGTGAACCGCATCGAGCTGAAGGGCATCGACTTCAAGGA 1600
AGAAGTTCCTGCTGCCGTTGATGTTCTGGGCGCGGCTCCACTTCAAGCTCCCGCTGTGGGACCACTTGGCGTAGCTCGACTTCCCGTAGCTGAAGTTCCT

140 145 150 155 160 165 170
F F K D D G N Y K T R A E V K F E G D T L V N R I E L K G I D F K E
eGFP-hSTIM1
EGFP

GGACGGCAACATCCTGGGGCACAAGCTGGAGTACAACTACAACAGCCACAACGTCTATATCATGGCCGACAAGCAGAAGAACGGCATCAAGGTGAACTTC 1700
CCTGCCGTTGTAGGACCCCGTGTTCGACCTCATGTTGATGTTGTCGGTGTTGCAGATATAGTACCGGCTGTTCGTCTTCTTGCCGTAGTTCCACTTGAAG

175 180 185 190 195 200
D G N I L G H K L E Y N Y N S H N V Y I M A D K Q K N G I K V N F
eGFP-hSTIM1
EGFP

AAGATCCGCCACAACATCGAGGACGGCAGCGTGCAGCTCGCCGACCACTACCAGCAGAACACCCCCATCGGCGACGGCCCCGTGCTGCTGCCCGACAACC 1800
TTCTAGGCGGTGTTGTAGCTCCTGCCGTCGCACGTCGAGCGGCTGGTGATGGTCGTCTTGTGGGGGTAGCCGCTGCCGGGGCACGACGACGGGCTGTTGG

205 210 215 220 225 230 235
K I R H N I E D G S V Q L A D H Y Q Q N T P I G D G P V L L P D N
eGFP-hSTIM1
EGFP

ACTACCTGAGCACCCAGTCCGCCCTGAGCAAAGACCCCAACGAGAAGCGCGATCACATGGTCCTGCTGGAGTTCGTGACCGCCGCCGGGATCACTCTCGG 1900
TGATGGACTCGTGGGTCAGGCGGGACTCGTTTCTGGGGTTGCTCTTCGCGCTAGTGTACCAGGACGACCTCAAGCACTGGCGGCGGCCCTAGTGAGAGCC

240 245 250 255 260 265 270
H Y L S T Q S A L S K D P N E K R D H M V L L E F V T A A G I T L G
eGFP-hSTIM1
EGFP

S1 Table. Continued

```
CATGGACGAGCTGTACAAGTCCGGCGCCAACTCTGAGGAGTCCACTGCAGCAGAGTTTTGCCGAATTGACAAGCCCCTGTGTCACAGTGAGGATGAGAAA  2000
GTACCTGCTCGACATGTTCAGGCCGCGGTTGAGACTCCTCAGGTGACGTCGTCTCAAAACGGCTTAACTGTTCGGGGACACAGTGTCACTCCTACTCTTT
275 280 285 290 295 300
M D E L Y K S G A N S E E S T A A E F C R I D K P L C H S E D E K
eGFP-hSTIM1
EGFP

CTCAGCTTCGAGGCAGTCCGTAACATCCACAAACTGATGGACGATGATGCCAATGGTGATGTGGATGTGGAAGAAAGTGATGAGTTCCTGAGGGAAGACC  2100
GAGTCGAAGCTCCGTCAGGCATTGTAGGTGTTTGACTACCTGCTACTACGGTTACCACTACACCTACACCTTCTTTCACTACTCAAGGACTCCCTTCTGG
305 310 315 320 325 330 335
L S F E A V R N I H K L M D D D A N G D V D V E E S D E F L R E D
eGFP-hSTIM1

TCAATTACCATGACCCAACAGTGAAACACAGCACCTTCCATGGTGAGGATAAGCTCATCAGCGTGGAGGACCTGTGGAAGGCATGGAAGTCATCAGAAGT  2200
AGTTAATGGTACTGGGTTGTCACTTTGTGTCGTGGAAGGTACCACTCCTATTCGAGTAGTCGCACCTCCTGGACACCTTCCGTACCTTCAGTAGTCTTCA
340 345 350 355 360 365 370
L N Y H D P T V K H S T F H G E D K L I S V E D L W K A W K S S E V
eGFP-hSTIM1
```

```
ATACAATTGGACCGTGGATGAGGTGGTACAGTGGCTGATCACATATGTGGAGCTGCCTCAGTATGAGGAGACCTTCCGGAAGCTGCAGCTCAGTGGCCAT  2300
TATGTTAACCTGGCACCTACTCCACCATGTCACCGACTAGTGTATACACCTCGACGGAGTCATACTCCTCTGGAAGGCCTTCGACGTCGAGTCACCGGTA
375 380 385 390 395 400
Y N W T V D E V V Q W L I T Y V E L P Q Y E E T F R K L Q L S G H
eGFP-hSTIM1

GCCATGCCAAGGCTGGCTGTCACCAACACCACCATGACAGGGACTGTGCTGAAGATGACAGACCGGAGTCATCGGCAGAAGCTGCAGCTGAAGGCTCTGG  2400
CGGTACGGTTCCGACCGACAGTGGTTGTGGTGGTACTGTCCCTGACACGACTTCTACTGTCTGGCCTCAGTAGCCGTCTTCGACGTCGACTTCCGAGACC
405 410 415 420 425 430 435
A M P R L A V T N T T M T G T V L K M T D R S H R Q K L Q L K A L
eGFP-hSTIM1

ATACAGTGCTCTTTGGGCCTCCTCTCTTGACTCGCCATAATCACCTCAAGGACTTCATGCTGGTGGTGTCTATCGTTATTGGTGTGGGCGGCTGCTGGTT  2500
TATGTCACGAGAAACCCGGAGGAGAGAACTGAGCGGTATTAGTGGAGTTCCTGAAGTACGACCACCACAGATAGCAATAACCACACCCGCCGACGACCAA
440 445 450 455 460 465 470
D T V L F G P P L L T R H N H L K D F M L V V S I V I G V G G C W F
eGFP-hSTIM1

TGCCTATATCCAGAACCGTTACTCCAAGGAGCACATGAAGAAGATGATGAAGGACTTGGAGGGGTTACACCGAGCTGAGCAGAGTCTGCATGACCTTCAG  2600
ACGGATATAGGTCTTGGCAATGAGGTTCCTCGTGTACTTCTTCTACTACTTCCTGAACCTCCCCAATGTGGCTCGACTCGTCTCAGACGTACTGGAAGTC
475 480 485 490 495 500
A Y I Q N R Y S K E H M K K M M K D L E G L H R A E Q S L H D L Q
eGFP-hSTIM1
```

```
GAAAGGCTGCACAAGGCCCAGGAGGAGCACCGCACAGTGGAGGTGGAGAAGGTCCATCTGGAAAAGAAGCTGCGCGATGAGATCAACCTTGCTAAGCAGG  2700
CTTTCCGACGTGTTCCGGGTCCTCCTCGTGGCGTGTCACCTCCACCTCTTCCAGGTAGACCTTTTCTTCGACGCGCTACTCTAGTTGGAACGATTCGTCC
505 510 515 520 525 530 535
E R L H K A Q E E H R T V E V E K V H L E K K L R D E I N L A K Q
eGFP-hSTIM1

AAGCCCAGCGGCTGAAGGAGCTGCGGGAGGGTACTGAGAATGAGCGGAGCCGCCAAAAATATGCTGAGGAGGAGTTGGAGCAGGTTCGGGAGGCCTTGAG  2800
TTCGGGTCGCCGACTTCCTCGACGCCCTCCCATGACTCTTACTCGCCTCGGCGGTTTTTATACGACTCCTCCTCAACCTCGTCCAAGCCCTCCGGAACTC
540 545 550 555 560 565 570
E A Q R L K E L R E G T E N E R S R Q K Y A E E E L E Q V R E A L R
eGFP-hSTIM1

GAAAGCAGAGAAGGAGCTAGAATCTCACAGCTCATGGTATGCTCCAGAGGCCCTTCAGAAGTGGCTGCAGCTGACACATGAGGTGGAGGTGCAATATTAC  2900
CTTTCGTCTCTTCCTCGATCTTAGAGTGTCGAGTACCATACGAGGTCTCCGGGAAGTCTTCACCGACGTCGACTGTGTACTCCACCTCCACGTTATAATG
575 580 585 590 595 600
K A E K E L E S H S S W Y A P E A L Q K W L Q L T H E V E V Q Y Y
eGFP-hSTIM1

AACATCAAGAAGCAAAATGCTGAGAAGCAGCTGCTGGTGGCCAAGGAGGGGGCTGAGAAGATAAAAAAGAAGAGAAACACACTCTTTGGCACCTTCCACG  3000
TTGTAGTTCTTCGTTTTACGACTCTTCGTCGACGACCACCGGTTCCTCCCCCGACTCTTCTATTTTTTCTTCTCTTTGTGTGAGAAACCGTGGAAGGTGC
605 610 615 620 625 630 635
N I K K Q N A E K Q L L V A K E G A E K I K K K R N T L F G T F H
eGFP-hSTIM1
```

S1 Table. Continued

TGGCCCACAGCTCTTCCCTGGATGATGTAGATCATAAAATTCTAACAGCTAAGCAAGCACTGAGCGAGGTGACAGCAGCATTGCGGGAGCGCCTGCACCG 3100
ACCGGGTGTCGAGAAGGGACCTACTACATCTAGTATTTTAAGATTGTCGATTCGTTCGTGACTCGCTCCACTGTCGTCGTAACGCCCTCGCGGACGTGGC
640 645 650 655 660 665 670
V A H S S S L D D V D H K I L T A K Q A L S E V T A A L R E R L H R
eGFP-hSTIM1

CTGGCAACAGATCGAGATCCTCTGTGGCTTCCAGATTGTCAACAACCCTGGCATCCACTCACTGGTGGCTGCCCTCAACATAGACCCCAGCTGGATGGGC 3200
GACCGTTGTCTAGCTCTAGGAGACACCGAAGGTCTAACAGTTGTTGGGACCGTAGGTGAGTGACCACCGACGGGAGTTGTATCTGGGGTCGACCTACCCG
675 680 685 690 695 700
W Q Q I E I L C G F Q I V N N P G I H S L V A A L N I D P S W M G
eGFP-hSTIM1

AGTACACGCCCCAACCCTGCTCACTTCATCATGACTGACGACGTGGATGACATGGATGAGGAGATTGTGTCTCCCTTGTCCATGCAGTCCCCTAGCCTGC 3300
TCATGTGCGGGGTTGGGACGAGTGAAGTAGTACTGACTGCTGCACCTACTGTACCTACTCCTCTAACACAGAGGGAACAGGTACGTCAGGGGATCGGACG
705 710 715 720 725 730 735
S T R P N P A H F I M T D D V D D M D E E I V S P L S M Q S P S L
eGFP-hSTIM1

AGAGCAGTGTTCGGCAGCGCCTGACGGAGCCACAGCATGGCCTGGGATCTCAGAGGGATTTGACCCATTCCGATTCGGAGTCCTCCCTCCACATGAGTGA 3400
TCTCGTCACAAGCCGTCGCGGACTGCCTCGGTGTCGTACCGGACCCTAGAGTCTCCCTAAACTGGGTAAGGCTAAGCCTCAGGAGGGAGGTGTACTCACT
740 745 750 755 760 765 770
Q S S V R Q R L T E P Q H G L G S Q R D L T H S D S E S S L H M S D
eGFP-hSTIM1

CCGCCAGCGTGTGGCCCCCAAACCTCCTCAGATGAGCCGTGCTGCAGACGAGGCTCTCAATGCCATGACTTCCAATGGCAGCCACCGGCTGATCGAGGGG 3500
GGCGGTCGCACACCGGGGGTTTGGAGGAGTCTACTCGGCACGACGTCTGCTCCGAGAGTTACGGTACTGAAGGTTACCGTCGGTGGCCGACTAGCTCCCC
775 780 785 790 795 800
R Q R V A P K P P Q M S R A A D E A L N A M T S N G S H R L I E G
eGFP-hSTIM1

GTCCACCCAGGGTCTCTGGTGGAGAAACTGCCTGACAGCCCTGCCCTGGCCAAGAAGGCATTACTGGCGCTGAACCATGGGCTGGACAAGGCCCACAGCC 3600
CAGGTGGGTCCCAGAGACCACCTCTTTGACGGACTGTCGGGACGGGACCGGTTCTTCCGTAATGACCGCGACTTGGTACCCGACCTGTTCCGGGTGTCGG
805 810 815 820 825 830 835
V H P G S L V E K L P D S P A L A K K A L L A L N H G L D K A H S
eGFP-hSTIM1

TGATGGAGCTGAGCCCCTCAGCCCCACCTGGTGGCTCTCCACATTTGGATTCTTCCCGTTCTCACAGCCCCAGCTCCCCAGACCCAGACACACCATCTCC 3700
ACTACCTCGACTCGGGGAGTCGGGGTGGACCACCGAGAGGTGTAAACCTAAGAAGGGCAAGAGTGTCGGGGTCGAGGGGTCTGGGTCTGTGTGGTAGAGG
840 845 850 855 860 865 870
L M E L S P S A P P G G S P H L D S S R S H S P S S P D P D T P S P
eGFP-hSTIM1

AGTTGGGGACAGCCGAGCCCTGCAAGCCAGCCGAAACACACGCATTCCCCACCTGGCTGGCAAGAAGGCTGTGGCTGAGGAGGATAATGGCTCTATTGGC 3800
TCAACCCCTGTCGGCTCGGGACGTTCGGTCGGCTTTGTGTGCGTAAGGGGTGGACCGACCGTTCTTCCGACACCGACTCCTCCTATTACCGAGATAACCG
875 880 885 890 895 900
V G D S R A L Q A S R N T R I P H L A G K K A V A E E D N G S I G
eGFP-hSTIM1
