# Supplementary material for: Detecting In-Situ oligomerization of engineered STIM1 proteins by diffraction-limited optical imaging
Source: PLoS One. 2019 Mar 25;14(3):e0213655. doi: 10.1371/journal.pone.0213655 (PMC6433367; doi:10.1371/journal.pone.0213655)
Supplement: S3 Table — A one-way ANOVA was performed using the raw data shown in Fig 6. (PDF) [file pone.0213655.s013.pdf]

$$H_0 : \mu_1 = \mu_2 = \mu_3 = \mu_4 = \mu_5 = \mu_6$$

$$H_1 : \quad \textit{Means are not equal}$$

About 900nm away from the PM

Number of clusters	24
Total number of molecular entities, N	78
Number of oligomeric species, k	6

	1-fold	2-fold	3-fold	4-fold	5-fold	6-fold	Grand Mean, $\overline{X}$
Counts, n_j	28	15	18	11	9	0	13.50
Counts/Cluster, $\overline{X}_j$	1.167	0.625	0.750	0.458	0.375	0	0.563

The test statistic*, F_{test} = 2.865 > F ~ 2.3 for $df_1 = 5; df_2 = 72$ at $\alpha = 0.05$ **REJECT H_0**

$$^{*}F_{test} = \frac{\sum n_j(\overline{X}_j - \overline{X})^2 / (k-1)}{\sum\sum(X - \overline{X}_j)^2 / (N-k)}$$
